# Lifestyle intervention to prevent type 2 diabetes after a pregnancy complicated by gestational diabetes mellitus: a systematic review and meta-analysis update

**DOI:** 10.1186/s13098-025-01606-x

**Published:** 2025-02-21

**Authors:** Paula Andreghetto Bracco, Angela Jacob Reichelt, Luísia Feichas Alves, Pedro Rodrigues Vidor, Maria Lúcia Rocha Oppermann, Bruce Bartholow Duncan, Maria Inês Schmidt

**Affiliations:** 1 Statistics Department, Universidade Federal do Rio Grande do Sul, Porto Alegre, Brazil; 2 Endocrinology and Metabolism Service, Hospital de Clínicas de Porto Alegre, Porto Alegre, Brazil; 3 Postgraduate Program in Epidemiology, Universidade Federal do Rio Grande do Sul, Porto Alegre, Brazil; 4 Central Library, Universidade Federal do Rio Grande do Sul, Porto Alegre, Brazil; 5 School of Medicine, Universidade Federal do Rio Grande do Sul, Porto Alegre, Brazil; 6 Department of Obstetrics and Gynecology, School of Medicine, Universidade Federal do Rio Grande do Sul, Porto Alegre, Brazil; 7 Hospital de Clínicas de Porto Alegre, Porto Alegre, Brazil

**Keywords:** Diabetes mellitus, Gestational diabetes, Lifestyle, Meta-analysis

## Abstract

**Background:**

Women with prior gestational diabetes mellitus (GDM) are at increased risk of type 2 diabetes, and lifestyle intervention (LSI) offered a decade after pregnancy is effective in preventing diabetes. However, since diabetes frequently onsets in the initial years following pregnancy, preventive actions should be implemented closer to pregnancy. We aimed to assess the effect of lifestyle interventions, compared to standard care, in reducing the incidence of diabetes following a pregnancy complicated by GDM.

**Methods:**

We searched the Cochrane Library, Embase, MEDLINE, and Web of Science from inception to July 21, 2024, to identify randomized controlled trials (RCTs) testing LSI to prevent diabetes following gestational diabetes. We followed the Preferred Reporting Items for Systematic Reviews and Meta-Analyses (PRISMA) guidelines. We evaluated the risk of bias with the Cochrane Collaboration Risk of Bias tool RoB-2 and the certainty of the evidence with GRADE methodology. We used the DerSimonian-Laird random effects pooling method and evaluated heterogeneity with the I^2^ statistic and the Chi^2^ test.

**Results:**

We identified 24 studies involving 9017 women. In studies without high risk of bias (18 studies; 8,357 women), LSI reduced the incidence of diabetes by 19% (RR = 0.81; 95%CI 0.71.0.93). The effect was significant and more protective (RR = 0.78; 0.65, 0.94) in studies evaluating women with GDM identified specifically as at a higher risk of diabetes, compared to those intervening on women with GDM irrespective of risk (RR = 0.85; 0.70, 1.04). Similarly, when expressed in absolute terms, the overall number needed to treat (NNT) was 56 considering all studies, 71 for women with GDM irrespective of risk, and 31 for women with GDM at high risk. The intervention produced a lower weight gain (mean difference=-0.88 kg;-1.52, -0.23 for all studies; -0.62 kg;-1.22, -0.02 for studies without high risk of bias). The effects were robust in sensitivity analyses and supported by evidence of moderate certainty for diabetes and weight change.

**Conclusions:**

LSI offered to women with GDM following pregnancy is effective in preventing type 2 diabetes, despite the small postpartum weight change. The impact of LSI on incidence reduction was greater for women with GDM at a higher diabetes risk.

**PROSPERO:**

Registration number CRD42024555086, Jun 28, 2024.

**Supplementary Information:**

The online version contains supplementary material available at 10.1186/s13098-025-01606-x.

## Background

The Global Burden of Disease Study estimated that 529 million people were living with diabetes worldwide in 2021, most of them presenting with type 2 diabetes [1]. The resulting burden of type 2 diabetes is high and increasing. Moreover, its rising prevalence among young adults – from 2.9 to 3.8% between 2013 and 2021, according to the 10th edition of the Diabetes Atlas [2] – is producing more severe and long-lasting cases [3].

Intensive lifestyle intervention (LSI) can prevent or delay the onset of type 2 diabetes when offered to high-risk subjects with an abnormal oral glucose tolerance test (OGTT) [4–6]. However, reaching out and performing the OGTT to identify high-risk individuals is challenging. In this regard, the routine use of an OGTT to screen for gestational diabetes mellitus (GDM) [7] in pregnancy is a great opportunity to identify high-risk subjects. GDM is found in 16.7% of pregnancies [2] and is associated with a nearly 10-fold increased risk of type 2 diabetes [8]. Intervening in this group of women offers an excellent opportunity for diabetes prevention. Indeed, a Diabetes Prevention Program (DPP) subgroup analysis of women with previous GDM who received LSI on average a decade after the index pregnancy found a ~ 50% decreased incidence of diabetes [9]. However, many women with GDM develop diabetes in the initial years following pregnancy. Thus, if effective, diabetes prevention interventions should start earlier, as soon as feasible, following a pregnancy with GDM.

To investigate the effectiveness of LSI offered closer to the index pregnancy, we systematically reviewed the literature in 2018, identifying eight studies (1647 women) with information regarding the incidence of type 2 diabetes. Heavily based on pilot studies or preliminary reports, our meta-analysis suggested a moderate decreased risk (RR = 0.75; 95% CI: 0.55–1.03), only statistically significant (RR = 0.61; 95% CI: 0.40–0.94) when LSI was initiated less than six months after pregnancy [10]. Subsequent systematic reviews, including additional studies, also found a moderately sized effect [11–14], though such analyses still included some studies with a high risk of bias.

Given the uncertainty about the actual size of the reduction in diabetes incidence with LSI offered after a pregnancy complicated by GDM, the discrepancies across reviews in the selection and analysis of studies, and the recent publication of many trials not included in previous reviews, a new and more comprehensive assessment of the available evidence is warranted.

We aimed to update our initial systematic review [10] by adding all new diabetes prevention trials following GDM pregnancies that informed incident cases of diabetes. Specifically, our objective was to synthesize the evidence for the effectiveness of LSI in reducing the incidence of type 2 diabetes when offered following a pregnancy complicated by GDM. Given the large number of women included, we also evaluated the robustness of the evidence through several sensitivity analyses.

## Methods

We adhered to the guidelines outlined in the Preferred Reporting Items for Systematic Reviews and Meta-Analyses (PRISMA) guidelines [15] and registered the protocol in the PROSPERO database on Jun 28, 2024 (CRD42024555086).

To assess the effectiveness of LSI following a pregnancy complicated by GDM, our primary outcome was the postpartum incidence of type 2 diabetes during the trials, and our secondary outcome was postpartum weight change assessed in studies reporting incident cases of type 2 diabetes.

### Data sources and search strategy

We searched for original articles in the Cochrane Library, Embase, PubMed (MEDLINE), and Web of Science from inception up to July 21st, 2024, with no language or publication time restrictions. Following the PICOS strategy (Population, Intervention, Comparator, Outcome, Study design), we searched articles with the terms “gestational diabetes, diabetes, lifestyle intervention, and randomized controlled trial.” As preconized in the Cochrane Handbook [16], to enhance sensitivity, we used indexed and free-text terms for randomized trials. This strategy captured some observational studies, which, when identified, were excluded. The complete search strategy for each database is detailed in Supplementary Table 1. One author (LFA) conducted the literature search, imported records into Zotero^®^ [17] for duplicate checking and removal, and imported the remaining records into Rayyan^®^ [18] for our screening.

### Eligibility criteria for study selection

Eligibility included: (1) women with a recent pregnancy complicated by GDM, irrespective of the diagnostic criteria, (2) who were offered postpartum lifestyle interventions (diet, exercise, breastfeeding, and other related interventions), (3) and compared to women receiving usual postpartum care (4) with assessment for the incidence of type 2 diabetes (5) within a randomized controlled trial. Consistent with our aim of assessing intervention following a pregnancy complicated by GDM, rather than in subjects with an abnormal OGTT, as in the classic diabetes prevention studies [9], we did not specify a cut-off for the number of years postpartum at randomization.

All authors screened eligible studies scrutinizing titles and abstracts in Rayyan^®^. At least two authors screened each record, and group discussions resolved discrepancies. At least two authors reviewed full-text articles of potentially eligible studies against the pre-specified inclusion criteria; group discussions resolved disagreements.

We excluded studies enrolling women with baseline diabetes, testing drug interventions, intervening only during pregnancy, and using a recruitment strategy not based on a previous diagnosis of GDM. After full-text scrutiny, we also excluded reports not informing incident cases of diabetes based on a wrong study population, with too short a follow-up (10 weeks), with an observational study design, or based on conference information not complemented by a published research protocol. When multiple reports on the same study were available, we retained only those providing relevant information.

To ensure the identification of all potentially eligible studies, we also reviewed the reference list of included trials and other systematic reviews on the subject.

### Data extraction

Data extraction was carried out using Excel^®^ software for Windows. Articles not written in English were read with the aid of automatic translators. Three authors (AJR, MLO, LFA), in pairs, extracted data on pre-defined items: (a) study identification (author, year of publication, registration, study name, country); (b) eligibility criteria; (c) type of trial design; (d) participant characteristics: maternal age and body mass index (BMI); (e) GDM diagnostic criteria; (f) intervention: starting time, mode, duration, and length of follow-up; (g) number of women randomized and analyzed for diabetes in each group; (h) number of new cases of diabetes in each group; (i) information on the adherence to the intervention; (j) participants weight (before and after the intervention for both groups) or weight change. After extraction, data were compared across each author pair and corrected as necessary.

When the incidence of diabetes or any other information was not found in the articles, we checked previous systematic reviews reporting contact with study authors and, if necessary, contacted study authors directly.

### Risk of bias assessment

Two independent groups of reviewers (AJR, MLO, PV and MIS, BBD, PB) assessed the risk of bias separately for each outcome using the revised Cochrane Collaboration Risk of Bias 2.0 (RoB-2) for individual or cluster-randomized studies, as appropriate [19]. After their independent evaluations, the two groups discussed disagreements until reaching a consensus. Studies were categorized as having a low or high risk of bias or presenting some concerns. We considered the study context to be women with recent GDM receiving lifestyle interventions at postpartum.

### Certainty of evidence

We evaluated the certainty (high, moderate, low, very low) of the evidence according to the Grading of Recommendations, Assessment, Development, and Evaluation (GRADE) methodology, based on our assessment of the risk of bias, imprecision, inconsistency, indirectness, and publication bias [16].

### Data analysis and synthesis

We defined the incidence of diabetes as the number of new cases of diabetes in trial participants based on the diabetes definitions used by the authors, which followed standard criteria. We defined weight change as the final weight (usually reported at ~ one year) minus the weight measured at the trial baseline. When the baseline was during pregnancy, we used the first postpartum weight. When the follow-up was considerably longer than one year, we used as the final weight the closest to one year.

For diabetes, we estimated risk ratios and risk differences using the DerSimonian-Laird random effects pooling method [20]. For one study [21] reporting different follow-up times between treatment groups, we estimated the number of incident cases from the reported annual incidence multiplied by the reported median length of follow-up time in each. For weight differences, we used an inverse variance random effects model. We derived standard deviations from standard errors or 95% confidence intervals (CIs) as needed. We evaluated heterogeneity in outcomes across studies with the I^2^ statistic and Cochran’s Q test. Subgroup differences were tested using the Q-test for between subgroup homogeneity, as provided in the R package ‘meta’ (R Foundation for Statistical Computing, Vienna, Austria).

We evaluated the robustness of our summary estimates through several sensitivity analyses. For the primary outcome, we separately assessed the estimates obtained from groups of studies with different risks of bias (all studies and only those with low risk, some concern, or high risk of bias). We also separately meta-analyzed studies with at least one year of follow-up. For weight change, we summarized estimates from studies with no serious risk of bias. For both outcomes, we additionally summarized effects using fixed effects models. Publication bias was assessed using the funnel plot with Egger’s test [22] and the trim and fill approach [23].

We used R Studio^®^ software (2024.04.2 Build 764, Posit Software; version 4.2.0) and the R package ‘meta’ (R Foundation for Statistical Computing, Vienna, Austria) for all statistical analyses.

## Results

**Fig. 1:**
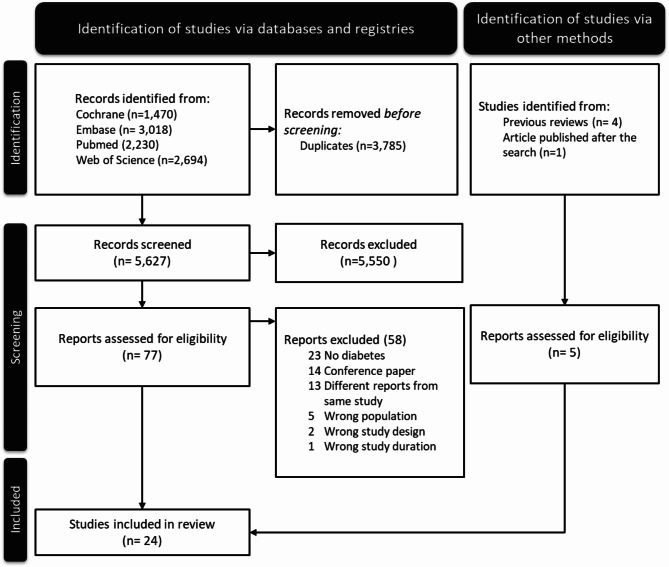
Flowchart for the identification of trials of lifestyle interventions offered to women following a pregnancy complicated by gestational diabetes that reported the incidence of type 2 diabetes

As seen in Fig. 1, after removing 3,785 duplicates, we obtained 5,627 records for title and abstract scrutiny. We excluded 5,550 records, leaving 77 reports for more detailed checking and evaluation, including full-text scrutiny. After excluding 58 reports for not presenting relevant data (Supplementary Table 2) and adding five reports (four identified from previous systematic reviews [24–27] and one recently published by three coauthors) [10], we identified a total of 24 studies with sufficient information to estimate the incidence of diabetes [21, 24–46].

### Description of studies

As seen in Table 1, the initial trial was published in 1999. After a decade, new trials were published almost yearly, ten during the last five years. Studies originated from most regions of the world -- five from Europe, four from American regions (three from North and one from South America), three from Oceania, and 12 from Asia. Half of the studies were from low- and middle-income countries. Although focusing our search on randomized trials, we did include one study that used historical controls to compare with the women receiving the experimental intervention. Three studies were cluster randomized trials, and 20 were individually randomized trials. There were six pilot or feasibility trials.

**Table 1:** Characteristics of studies evaluating a lifestyle intervention program for the prevention of type 2 diabetes after a pregnancy complicated by gestational diabetes mellitus (GDM)

Author, year (Country)	Design	Selection of GDM or higher risk GDM		Intervention			Primary outcome
*Study name* *Registry*	Sample size		Experimental group	Control group	Starting time	Duration	
Wein et al., 1999 (Australia)	RCT *N* = 200	GDM + postpartum IGT	3-monthly phone calls (intensive diet)	Diet and/or PA	Shortly after pregnancy	4 years	Diabetes and IGT
Cheung et al., 2011 (Australia)	Pilot RCT *N* = 43	GDM + physical inactivity	1 individual session on-site, phone calls 2, 6, 10 weeks. Maintenance: phone calls at 26 and 34 weeks + messages	Diet and/or PA + courtesy call at 6 months	< 4 years after pregnancy	3 months	PA improvement (diabetes as a secondary outcome)
Ji et al., 2011 (China)	RCT *N* = 144	GDM + postpartum IGT/IFG	6 home visits, phone calls (diet + PA)	Prevention education + reminder to revisit at 6 months postpartum	0 -12weeks after pregnancy	4 months	Weight, HbA1c and OGTT (diabetes not specified as primary outcome)
Yu et al., 2012 (China)	RCT *N* = 134	GDM + postpartum IGT/ IFG	3-monthly individual sessions, phone calls (diet + PA)	Health promotion materials	6–8 weeks after pregnancy	2 years	Insulin resistance and ß-cell dysfunction (diabetes not specified as primary outcome)
Shyam et al., 2013 (Malaysia) *Malaysian National Medical Research - ID: 5183*	RCT *N* = 77	GDM + postpartum IGT/ IFG	1 individual in-site session, e-mail or SMS, by-weekly (low GI diet + PA)	Diet and/or PA	≥ 2 months after pregnancy	6 months	FPG and 2 h glucose (diabetes not specified as primary outcome)
Guo et al., 2013 (China)	RCT *N* = 100	GDM	Phone calls, individual sessions (diet + PA)	No postpartum behavioral intervention	NR	1 year	Diabetes
Geng et al., 2014 (China)	Feasibility trial, historic controls *N* = 100	GDM	Phone calls: 2x/week 1st month, then weekly (diet + PA)	Routine health education on the discharge day, no strict follow-up	4–6 weeks after pregnancy	1 year	Diabetes
Nicklas et al., 2014 (USA) *Balance after baby NCT01158131*	Feasibility RCT *N* = 75	GDM	Web-based program (12 modules) e/mail/web contact (weekly for 12 weeks; by-weekly up to 24 weeks, monthly thereafter) (diet + PA)	Handout at recruitment	6 weeks after pregnancy	1 year	Weight (diabetes not specified as primary outcome)
Shek et al., 2014 (China)	RCT *N* = 450	GDM + postpartum IGT	Individual sessions (2 first 3 months; 6 monthly thereafter (diet + PA)	No treatment. Follow-up as for the intervention group	6–8 weeks after pregnancy	3 years	Diabetes
Pérez-Ferre et al., 2015 (Spain) *ISRCTN24165302*	RCT *N* = 260	GDM	Group and individual sessions for 10 weeks (3 to 6 months postpartum) (Mediterranean diet + PA)	Mediterranean diet pattern	7–12 weeks after pregnancy	3 years	Diabetes
Ferrara et al., 2016 (USA) *GEM* *NCT01344278*	Cluster trial *N* = 2,280	GDM	Structured sessions by phone: 13 sessions guided by a book (6 weeks-6 months). Maintenance (7–12 months): 3 newsletters (diet + PA)	Letter to be screened for DM; diet + PA	During pregnancy	1 year	Weight (diabetes as an exploratory outcome)
Zilberman-Kravits et al., 2018 (Israel) *NCT 01480895*	RCT *N* = 180	GDM	3 individual sessions, 2–4 group meetings, onsite demonstration of physical activity and meals (diet + PA)	One individual education session on DM prevention	3–4 months after pregnancy	2 years	HOMA-IR (diabetes not specified as primary outcome)
McManus et al., 2018 (Canada) *Families defeating diabetes (FDD)* *NCT01425645*	RCT *N* = 170	GDM + overweight	Seminars, website, by-weekly emails, invitation to weekly walks (healthy living: diet + PA + breastfeeding)	Hard copy of the postpartum healthy living handout	NR	1 year	BMI, HbA1c, waist, weight (diabetes not specified as primary outcome)
Cheung et al., 2019 (Australia) *Smart Mums With Smart Phones ACTRN12616000067471*	Pilot RCT *N* = 60	GDM + insulin treatment in pregnancy	4x/weekly messages at postpartum; 2 diet counseling sessions (one face-to-face in pregnancy, one by phone at randomization (diet + PA)	Booklet on healthy lifestyle advice	During pregnancy	6 months	Post-partum OGTT testing (diabetes not specified as primary outcome)
Hu et al., 2022 (China) *Tianjin Gestational Diabetes Mellitus Prevention Program NCT01554358*	RCT *N* = 1,180	GDM + postpartum IGT/ IFG	6 face-to-face meetings (1st year), 2 sessions on-site and 2 phone calls/year up to 4.5 years (diet + PA)	Education on healthy lifestyle and DM prevention	~ 4–5 years after pregnancy (mean 27 months)	2 years	Diabetes, cardiovascular risk
Tandon et al., 2022 (India, Bangladesh, Sri Lanka) *LIVING* *NCT03305939*	RCT *N* = 1,612	GDM	4 face-to-face sessions (6 months) + 2 additional face-to-face sessions when needed, monthly phone calls, text messages during the intervention (diet + PA)	Referred to usual doctor for ongoing management	4.8–8.2 months after pregnancy	1 year	Diabetes/IGT
Potzel et al., 2022 (Germany) *TRIANGLE DRKS00012996*	RCT *N* = 66	GDM	Smartphone-delivered intervention through an app (diet + PA)	Flyer on lifestyle changes for DM prevention + psychosocial well-being and sleep themes as usual care	3–18 months after pregnancy	6 months	Reaching goals (nutritional aspects, PA, weight) (diabetes not specified as primary outcome)
Parsons et al., 2022 (United Kingdom) *GODDESS* *ISRCRN 52,675,820*	Feasibility trial *N*= 50	GDM or HbA1c ≥ 6% + overweight	Whatsapp group, PA app, e-support, 4 motivational interviews (diet + PA)	Email or text message with postpartum OGTT result; referred to usual doctor. At the end of the study, a support session	During pregnancy	6 months	Achieving weight change (diabetes not specified as primary outcome)
Lee et al., 2022 (Malaysia) *NMRR-15-2000-28718*	Cluster trial * N* = 650	GDM	6 individual sessions: one in pregnancy, one session at 6 weeks postpartum, 4 additional twice-year sessions (diet + PA)	Standard treatment + group therapy on diet and PA during the antenatal period	During pregnancy	2 years	Diabetes
Quansah et al., 2023 (Switzerland) *MySweetheart trial* *NCT02890693*	RCT *N* = 211	GDM	4 individual visits in pregnancy, 4 individual visits postpartum (multidisciplinary), up to 3 months, monthly telemedicine up to 1 year (diet + PA)	At 6–8 weeks and at 1 year postpartum: OGTT + clinical visit to advise on lifestyle changes based on cardio-metabolic laboratory results	During pregnancy	1 year	Weight, depression (diabetes not specified as primary outcome)
Iqbal et al., 2024 (Pakistan) *ISRCT N1138 7113*	Feasibility trial *N* = 180	GDM	Initial home session, one consultation at 1 month, reinforcement sessions at 3, 6, and 9 months, text messages 3x/week (diet + PA)	Usual advice from their care providers	6 weeks after pregnancy	1 year	Behavior and metabolic changes (diabetes not specified as primary outcome)
Minschart et al., 2024 (Belgium) *MELINDA* *NCT03559621*	RCT *N* = 240	GDM + postpartum IGT/IFG	1 individual face-to-face session, monthly phone sessions, smartphone app (diet + PA)	Referred to primary care for follow-up in line with normal routine	6–16 weeks after pregnancy	1 year	Reaching weight goals (diabetes as secondary outcome)
Sundarapperuma et al., 2024 (Sri Lanka) *SLCTR/2015/021*	Cluster trial *N* = 100	GDM	Individual face-to-face sessions: home visits monthly, bi-weekly phone calls (diet + PA)	Diet diary for 3 days/week; activity diary; 7-day pedometer use each month for the entire study; biweekly reminder to maintain activity and diet diaries	6 weeks after pregnancy	1 year	Trajectory to diabetes (diabetes not specified as primary outcome)
Schmidt et al., 2024 (Brazil) *LINDA-Brasil* *NCT02327286*	RCT *N* = 474	GDM + postpartum IGT/IFG and/or drug treatment for GDM during pregnancy	1 individual session, 20 telephone sessions guided by a book (diet + breastfeeding + PA)	Booklet on diet + PA + breastfeeding + advice on checking DM status	2 months after pregnancy	2 years	Diabetes

BMI: body mass index; FPG: fasting plasma glucose; HbA1c: glycated hemoglobin; HOMA-IR: homeostatic model assessment-insulin resistance; IGT: impaired glucose intolerance; IFG: impaired fasting glucose; NR: Not reported; OGTT: oral glucose tolerance test; PA: physical activity; RCT: randomized controlled trial

The diagnostic criteria for GDM varied. Five studies [35, 39, 40, 44, 45] used the International Association of Diabetes and Pregnancy Study Groups (IADPSG) criteria [47], three [33, 42, 43] the World Health Organization criteria [48], three [25, 30, 38] the Carpenter & Coustan criteria [7], three other criteria [36, 37, 41], and 10 did not specify the criteria used.

In nine studies, eligibility criteria required participants not only to have GDM, but to have an additional indication of being at higher risk for type 2 diabetes: having postpartum intermediate hyperglycemia (seven studies) and/or requiring medication to treat gestational diabetes (two studies).

Most studies intervened on diet and physical activity (22 studies), one only on diet and one only on physical activity, with variable delivery modes including onsite and at-home individual and group sessions, telephone sessions/chats, apps, or web-based platforms. Although interventions were predominantly delivered after pregnancy, generally starting soon after the postpartum glycemic reevaluation (less than two years), in five, they began during pregnancy, and in two, they allowed initiation up to four to five years after pregnancy. Interventions were not intensive and lasted from four months to four years.

The control group also received some degree of lifestyle intervention considered necessary by trial investigators, the intensity of which increased in more recent trials. This intervention included, among others, management by clinicians outside of the trial and written materials.

The incidence of diabetes was the primary outcome in nine studies. In the remaining studies, diabetes was a secondary or exploratory outcome or, though not listed as an outcome, was reported when new cases were detected. In five studies, the assessment of diabetes was done over less than one year of follow-up, in the remaining over approximately one year (10 studies), two years (four studies), or three or more years (five studies).

### Risk of bias

A detailed description of the risk of bias assessments is provided in the Supplementary Tables 3 and 4 and Supplementary Figs. 1 and 2.

For the incidence of diabetes, we evaluated 24 studies, six we judged as having a high risk of bias [27, 29, 34, 37, 44, 46], six as having a low risk of bias [21, 30, 32, 41, 42, 45], and the remaining 12 studies as presenting some concern. Among high-risk bias studies, the high risk arose from the randomization process in three, was due to deviations from the intended intervention in two, and was due to missing outcome data in four. The 12 studies with some bias concerns presented less severe problems in one or more of the above three dimensions.

For the weight change outcome, we gathered results from 16 studies, five of which had a high risk of bias. Among the 11 studies without a high risk of bias, we considered four to be of some concern and seven to have a low risk of bias. The risk of bias we identified regarding the outcome weight change occurred in the exact dimensions of those found in the articles on the incidence of diabetes.

### Effect of LSI on the incidence of diabetes

The 24 studies analyzed (9017 women) provided 762 incident cases of diabetes over an average follow-up time of 19.4 months. LSI reduced the incidence of diabetes by 24% (RR = 0.76; 95% CI 0.65–0.89).

**Fig. 2:**
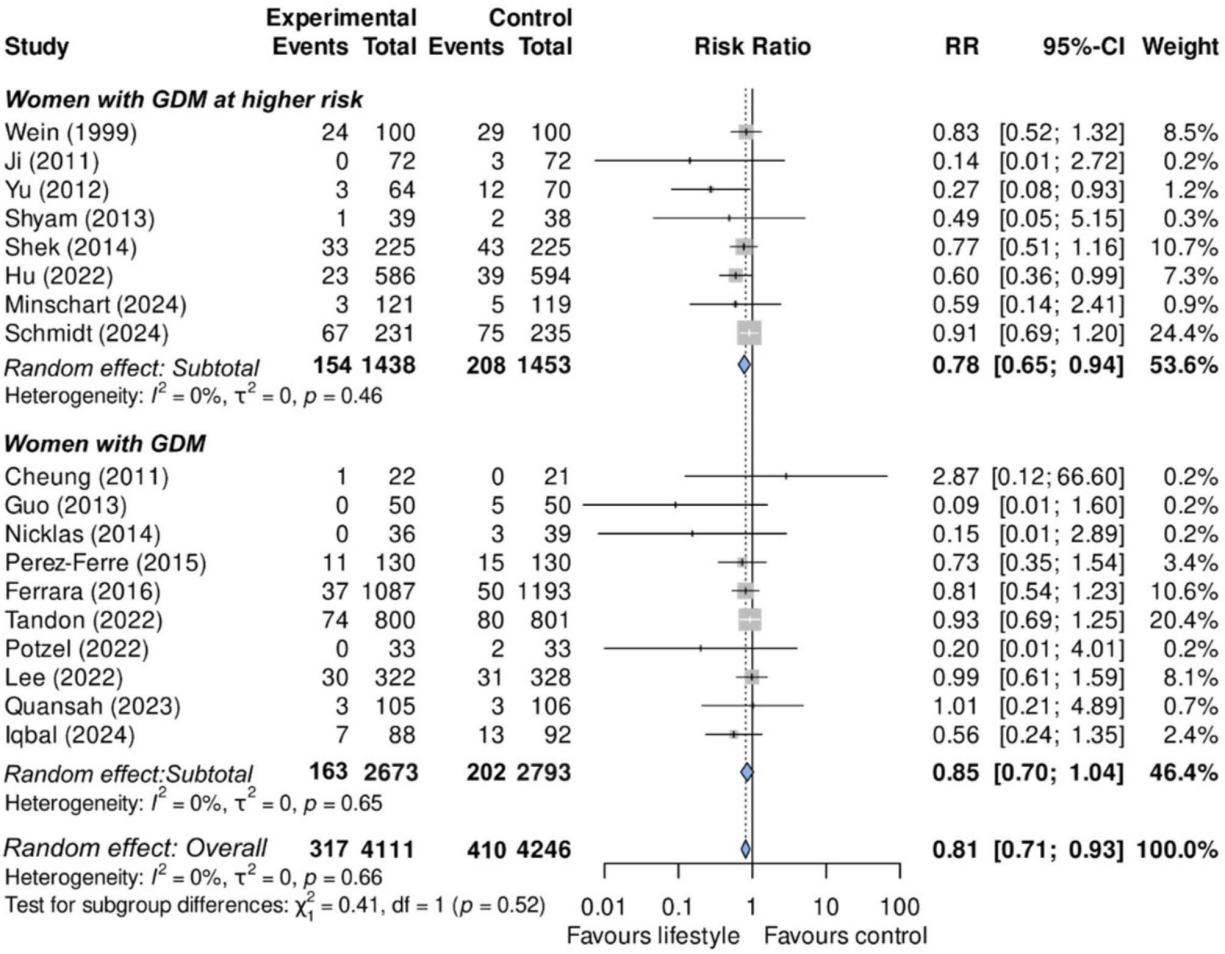
Effectiveness (risk ratios) of a lifestyle intervention program to prevent type 2 diabetes after a pregnancy complicated by gestational diabetes in the 18 studies without high risk of bias. Top: Women with GDM at higher risk. Bottom: Women with GDM. *Estimated from the annual risk in each group and multiplied by the study median length of follow-up

To enhance certainty, we did not include the six studies with a high risk of bias in the analyses producing our main results. As seen in Fig. 2, of the remaining 18 studies (8,357 women, 727 incident cases), all but two favored LSI. Individually, most studies did not achieve statistical significance, with effects ranging from RR = 0.09 (0.01, 1.60) to RR = 2.87 (0.12, 66.60). Over an average follow-up time of 21.8 months, LSI reduced the incidence of diabetes by 19% (RR = 0.81; 0.71, 0.93). The effect was numerically higher (*p* = 0.52) in the eight studies selecting women with GDM having a particularly higher risk of type 2 diabetes (RR = 0.78; 0.65, 0.94), compared to studies selecting all women with GDM, irrespective of diabetes risk (RR = 0.85; 0.70, 1.04).

**Fig. 3:**
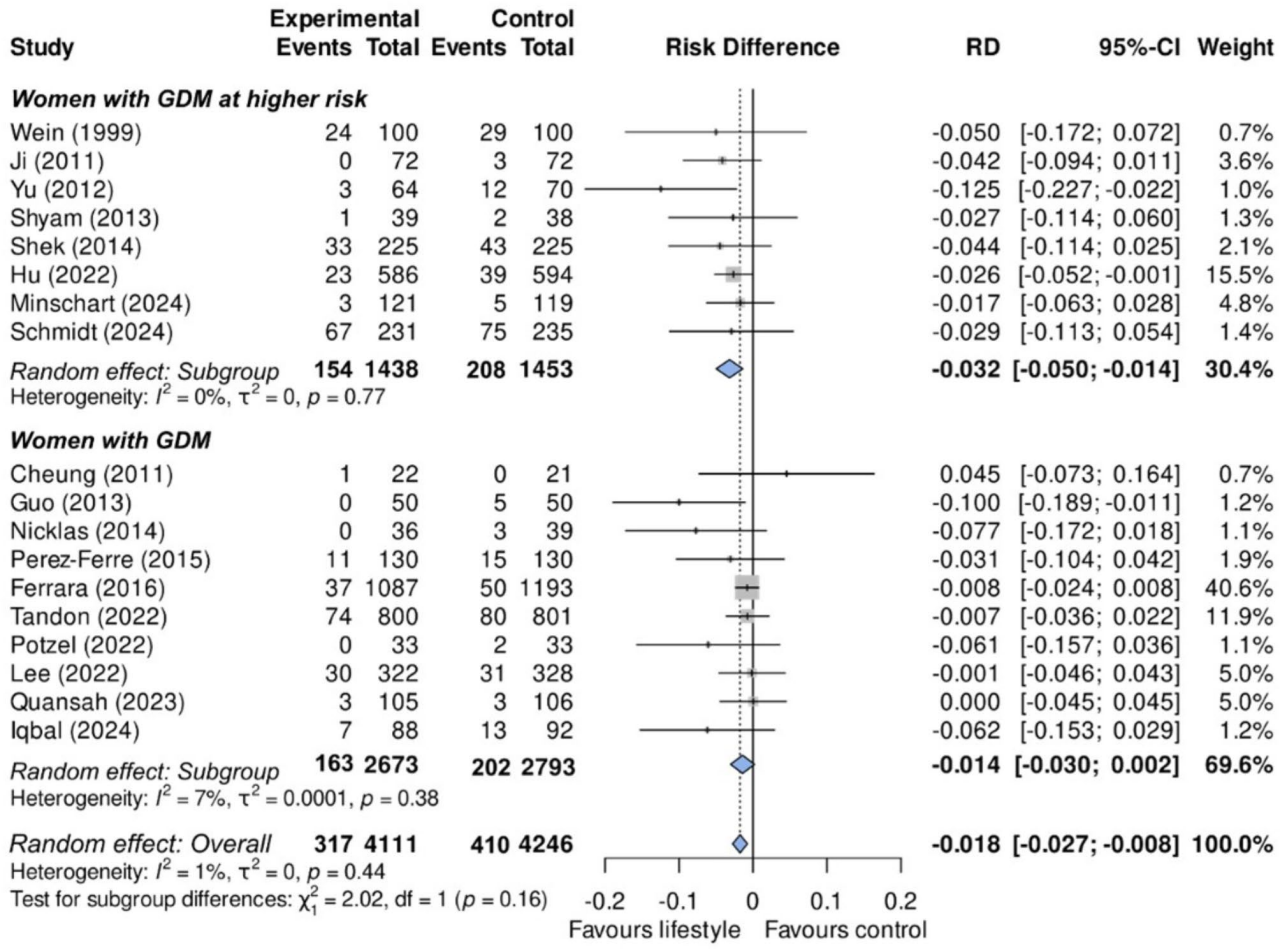
Effectiveness (risk differences) of a lifestyle intervention program to prevent type 2 diabetes after a pregnancy complicated by gestational diabetes in the 18 studies without high risk of bias. Top: Women with GDM at higher risk. Bottom: Women with GDM. *Estimated from the annual risk in each group and multiplied by the study median length of follow-up

Figure 3 shows the overall absolute risk difference for the 18 studies (RD = − 0.018 (95% CI -0.027; -0.008), translating into an NNT of 56. However, in studies for women with GDM with a particularly high risk of type 2 diabetes, we found a numerically larger (*p* = 0.16) effect (RD = − 0.032; 95% CI -0.050, -0.014), translating into an NNT of 31. These findings contrasted with the non-significant risk difference found (RD = − 0.014; 95% CI -0.030, 0.002; NNT = 71) in studies of women with GDM, irrespective of risk.

### Effect of LSI on postpartum weight change

**Fig. 4:**
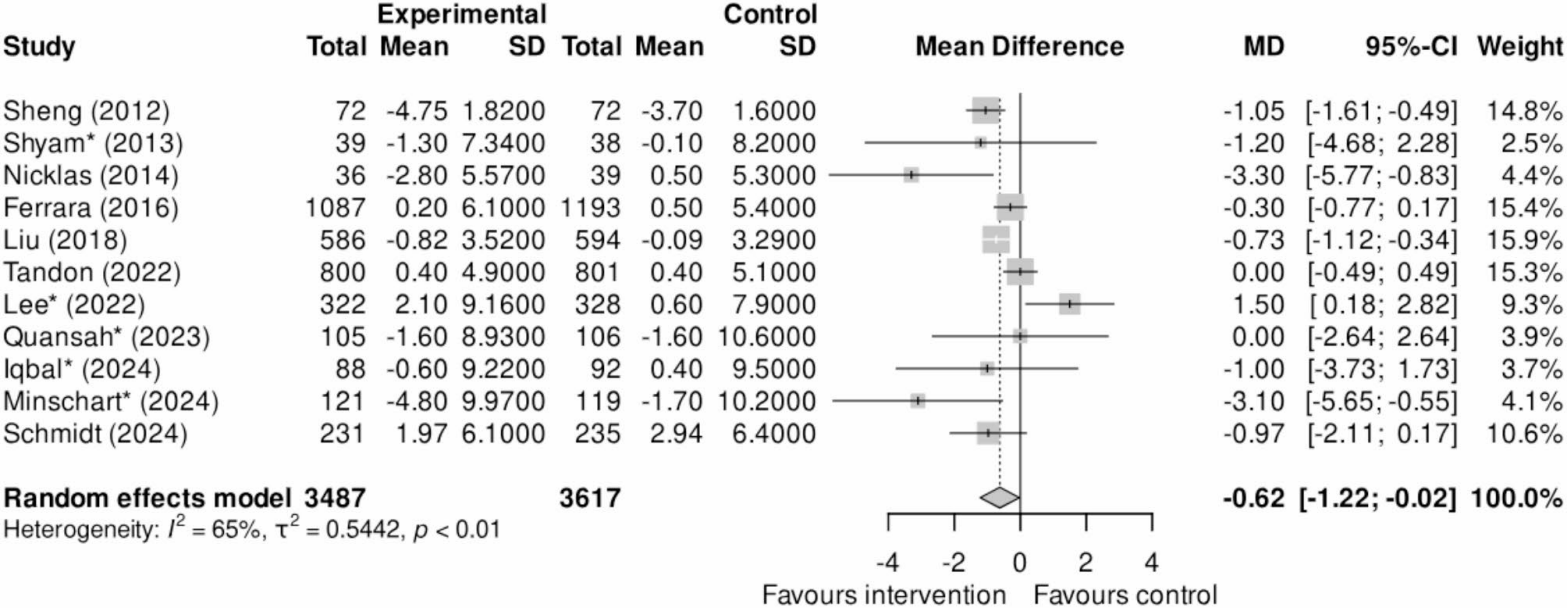
Effectiveness (weight difference, kg) of a lifestyle intervention program to prevent type 2 diabetes after a pregnancy complicated by gestational diabetes in the 11 studies without high risk of bias

For two studies, there was no information to assess weight change in the publication reporting the incidence of diabetes, and we extracted it from other publications of these studies [49, 50]. As seen in Fig. 4, the effect of LSI on postpartum mean weight change was assessed in 16 studies having the appropriate information. Differences in weight varied from 4.4 kg less to 3.4 kg greater weight gain with LSI in the individual studies. With an average follow-up time of 10.5 months, the overall summary difference was − 0.88 kg (-1.52, − 0.23), I^2^ = 66%.

When we excluded the five studies with a high risk of bias, the overall mean difference in the remaining 11 studies after 11.5 months of follow-up was − 0.62 kg (-1.21; − 0.02), I^2^ = 65%.

### Sensitivity analyses

As seen in Table 2, the protective effect in reducing the incidence of diabetes decreased as the quality of studies increased. The six studies with a high risk of bias had the greatest protection (RR = 0.19; 95% CI 0.08–0.50), and the six studies with a low risk of bias had the lowest protection, which was borderline statistically significant (RR-0.86; 0.73, 1.00). In an additional analysis, studies having at least one year of follow-up had a somewhat larger protective effect (RR = 0.73; 0.59, 0.90). Finally, the effects found with fixed effect models were similar to those found using random effect models. Thus, our main finding of an RR = 0.81 (95% CI 0.71, 0.93) in the 18 studies without high risk of bias was robust in these various sensitivity analyses.

**Table 2:** Sensitivity analyses of the effectiveness of lifestyle interventions to prevent type 2 diabetes after a pregnancy complicated by gestational diabetes

Incidence of diabetes	Studies	Participants	Cases	Effect		I^2^
	N	N	N	RR	95% CI	
Random effects						
All studies	24	9,017	762	0.76	0.65–0.89	8.1%
High risk of bias excluded	18	8,357	727	0.81	0.71–0.93	0.0%
Risk of bias assessment						
Only high risk	6	660	35	0.20	0.08–0.50	0.0%
Only “some concern”	12	3180	195	0.68	0.52–0.91	0.0%
Only low risk	6	5177	532	0.86	0.73-1.00	0.0%
≥1 year follow-up	19	8,620	751	0.73	0.59–0.90	19.2%
High risk of bias excluded	15	8,070	719	0.82	0.72–0.94	0.0%
Fixed effects						
All studies	24	9,017	762	0.74	0.65-085	8.1%
High risk of bias excluded	18	8,357	727	0.79	0.69–0.91	0.0%

Difference in weight change (kg)	Studies	Participants		Effect*		
	N	N		Mean	95% CI	
Random effects						
All studies	16	7664		-0.88	-1.52; -0.23	65.7%
High risk of bias excluded	11	7104		-0.62	-1.22; -0.02	65.0%
Only low risk	7	4890		-0.43	-0.93; 0.07	33.5%
Fixed effects						
All studies	16	7664		-0.57	-0.78; -0.35	65.7%

RR = relative risk. CI = confidence interval. * A negative number indicates a lesser mean weight gain in the experimental group

Although evaluated in a lower number of studies, the effect of weight change was also robust in various sensitivity analyses, the mean difference being somewhat lower in studies without a high risk of bias.

### Publication bias

A funnel plot (Supplementary Fig. 3) showed asymmetry in estimating the effects of LSI on the incidence of diabetes (Egger’s test *p* = 0.01). However, trim and fill analyses adding six hypothetical studies to compensate for the six asymmetric studies identified by the plot resulted in little change in the effect size (RR = 0.83; 95% CI 0.72, 0.96), demonstrating that the asymmetric, small-sized studies included in the meta-analysis caused only a minimal imbalance.

### Certainty of the evidence

In Supplementary Table 5 we present the evaluation of the quality of the evidence using the GRADE system.

The evidence for the incidence of diabetes outcome based on the 18 studies without high risk of bias encompassed 8,357 women and 727 events. Starting from high certainty, as defined by the studies with randomized design, we lowered certainty to moderate, given that 12 of the 18 studies presented some concerns in our evaluation of the risk of bias. No reduction was necessary for heterogeneity, imprecision, indirectness, or publication bias.

For weight change, the evidence provided was based on the 11 studies that did not present a high risk of bias, encompassing 7,104 women. Starting from high certainty, as defined by the studies’ randomized design, we lowered certainty to moderate due to heterogeneity. No reduction was necessary for risk of bias, imprecision, indirectness, or publication bias.

## Discussion

### Summary of findings

In this comprehensive review of diabetes prevention trials following a pregnancy complicated by GDM, LSI, compared to usual care, reduced the incidence of diabetes by 24% (24 trials, 9,017 women, 762 incident cases), with a weight gain of 0.88 kg less. With the exclusion of six trials found to have high risk of bias, incidence reduction was slightly less, 19% (18 trials, 8,357 women, 727 incident events), with an accompanying 0.62 kg lesser weight gain. Of note, in the eight trials evaluating women with GDM having an exceptionally higher risk of diabetes (presence of intermediate hyperglycemia at postpartum and/or use of pharmacologic treatment during pregnancy), the reduction in incidence was greater in both relative (22%) and absolute (NNT = 31) terms.

### Context

Our 19% (no high risk of bias (18 studies) to 24% (all studies) estimates of the reduction in diabetes incidence lie within the range of effects (11–43%) of previous systematic reviews [10–14, 51] assessing the impact of LSI in women following a pregnancy complicated by GDM. These effects are lower than the ~ 50% reported in the DPP subgroup analysis of women treated ~ 10 years after GDM [9]. They are somewhat closer to the effect size observed in real-world trials in the general population (29%) [52].

The effect on weight change we found (-0.62 to -0.88 kg) is lower than that found in a meta-analysis of studies aiming primarily at postpartum weight reduction with dietary and physical activity interventions in women with previous gestational diabetes (-2.19; 95% CI − 0.39, -0.98 kg) [53]. Although these trials illustrate that more intensive weight-reducing interventions are feasible at postpartum, it is reassuring that even the less than one kg weight difference we found was associated with a clinically relevant reduction in the incidence of diabetes in women with a recent GDM.

### Interpretation of diabetes incidence reduction

A few reasons may explain this lower effect following pregnancy. First, LSI at postpartum was usually less intensive compared to that offered in the classic trials in adults selected for having IGT. Second, women at postpartum are less likely to adhere to LSI due to the stress and the competing priorities of motherhood in this period. Third, GDM may represent a prodrome of a more severe form of diabetes found in young adults (< 40 years), whose pathophysiology [54] produces cases of earlier onset, which thus would not have been captured by the classic studies on diabetes prevention. Fourth, with increasing knowledge on the effectiveness of diabetes prevention over time, the usual care of women with GDM at postpartum is likely to have placed more emphasis on preventive actions. As such, the comparison “usual care” groups in our systematic review of post-GDM trials generally included more detailed preventive actions, particularly the more recent ones (Table 1), than the control group of classic trials conducted in the late 1990s. The DPP, for example, included, after randomization, one individual 20–30 min session on healthy lifestyle for diabetes prevention (diet, physical activity, weight loss goals, alcohol avoidance, and advice to stop smoking), with annual reinforcement. Finally, the COVID-19 pandemic may have negatively impacted the more recent trials. Although the magnitude of this effect is unknown, it may have decreased the associations found, as one study comparing the effects obtained before and during the pandemic indicated numerically lower benefits during the pandemic [41].

### Interpretation of the postpartum weight change

The small effect of LSI in postpartum weight change merits discussion. The intensity of the interventions was generally less than that implemented in the classic studies not following a pregnancy complicated by GDM [4, 5]. In addition, different from the expected decrease in postpartum weight in the general population, women with prior GDM frequently retained [55] or gained [56] weight postpartum. The reasons behind this are not clear. One might stem from the need to adopt intensive dietary interventions to control GDM during pregnancy, frequently accompanied by insulin or other types of diabetes medication. After pregnancy, the strict, usually weekly, surveillance ceases, leaving women freer to self-manage a somewhat abstract disease risk. Focusing on diabetes prevention can be overwhelmed by existing barriers [57], notably the concrete demands of recent motherhood. If this is the case, as we believe it to be, support for lifestyle changes is not only helpful, but it is essential to prevent postpartum weight gain and reduce diabetes and cardiovascular risk [58]. With the advances in information technology and the increased access to the Internet, designated apps can complement health professional supervision.

### Limitations

Some limitations of our systematic review must be considered. First, when assessing the risk of bias in LSI trials after pregnancy, some decisions were necessarily arbitrary, particularly those related to the deviations from the intended intervention due to the trial context and the seriousness of the incomplete outcome assessment. Second, we encountered limitations in the quality of the information extracted from the reports obtained for some studies. This was particularly true regarding the automated translations of text and tables from articles published in Chinese, which sometimes produced dubious findings. Third, for one study, we relied on a published summary of a medical conference presentation to obtain diabetes incidence.

Finally, we could only assess the impact of LSI with intention-to-treat (ITT) analysis since we found no studies doing per-protocol analysis. When analyzed appropriately, ITT and per-protocol approaches are valid [59]. Given the importance of preventing diabetes in the postpartum period, in which losses and partial adherence are expected to be large, the scarcity of per-protocol analyses is a significant limitation. Such analyses could inform the benefit of the interventions for women who embrace them.

### Strengths and contributions

Our review has considerable strengths and contributions. It is the most extensive review, providing a comprehensive, precise, and robust summary of effects. It is also the first meta-analysis permitting a statistically significant summary effect in studies with a low risk of bias. Finally, our findings support prioritizing women identified to be at higher risk for early intervention in the postpartum period. This strategy may provide a more feasible and effective approach to prevention.

### Generalizability and applicability

We included studies from most regions of the world. Although studies were evaluated as efficacy trials based on ITT analyses, most can be seen as close to real-world settings. Thus, we believe these results apply to most clinical settings.

### Further research

Although effective and presenting a satisfactory NNT of 31 when implemented for women with high-risk GDM, the less intensive lifestyle interventions offered in these trials could be further tailored [60]. Uptake and maintenance can be facilitated using websites and apps to enhance adherence, thus providing more significant benefits and extending the application to a greater fraction of women following a pregnancy complicated by GDM.

## Conclusion

Evidence with a moderate degree of certainty confirms that LSI offered at postpartum to women with GDM is effective in preventing type 2 diabetes, particularly for those at a higher risk of developing type 2 diabetes in the initial years after pregnancy. The reduction in the accompanying weight gain was small and provided moderate certainty evidence. Further research is needed to refine the mode of the interventions to gain greater benefits and applicability, possibly including medications when appropriate.

## Electronic supplementary material

Below is the link to the electronic supplementary material.

Supplementary Material 1

## Data Availability

Data are available upon reasonable request from PAB.

## Abbreviations

CI: Confidence interval
COVID: Coronavirus disease
DM: Diabetes mellitus
DPP: Diabetes Prevention Program
GDM: Gestational diabetes mellitus
GRADE: Grading of Recommendations, Assessment, Development, and Evaluation
BMI: Body mass index
FPG: Fasting plasma glucose
HbA1c: Glycated hemoglobin
HOMA-IR: Homeostatic model assessment-insulin resistance
IADPSG: International Association of Diabetes and Pregnancy Study Groups
IGT: Impaired glucose intolerance
IFG: Impaired fasting glucose
LSI: Lifestyle intervention
N: Number
NNT: Number needed to treat
NR: Not reported
OGTT: Oral glucose tolerance test
PA: Physical activity
PRISMA: Preferred Reporting Items for Systematic Reviews and Meta-Analyses
RCT: Randomized controlled trial
RD: Risk difference
RoB: Risk of bias
RR: relative risk
