# Supplementary material for: Lifestyle intervention to prevent type 2 diabetes after a pregnancy complicated by gestational diabetes mellitus: a systematic review and meta-analysis update
Source: Diabetol Metab Syndr. 2025 Feb 21;17:66. doi: 10.1186/s13098-025-01606-x (PMC11844165; doi:10.1186/s13098-025-01606-x)
Supplement: Supplementary file 1 — Supplementary Material 1 [file 13098_2025_1606_MOESM1_ESM.docx]

**Supplementary material**

[Supplementary Table 1. Search strategies at each database. 2](#_Toc181280210)

[Supplementary Table 2. Excluded references after full text screening. 5](#_Toc181280211)

[Supplementary Table 3. Risk of bias assessment for the incidence of diabetes in women with recent gestational diabetes. 9](#_Toc181280212)

[Supplementary Table 4. Risk of bias assessment for the weight change in women with recent gestational diabetes. 17](#_Toc181280213)

[Supplementary Table 5. Evaluation of the quality of the evidence using the GRADE system. 23](#_Toc181280214)

[Supplementary Figure 1. Risk of bias assessment for the incidence of diabetes in women with recent gestational diabetes. 24](#_Toc181280215)

[Supplementary Figure 2. Risk of bias assessment for the weight change in women with recent gestational diabetes. 25](#_Toc181280216)

[Supplementary Figure 3. Publication bias. 26](#_Toc181280217)

# Supplementary Table 1. Search strategies at each database.

*Database: Cochrane Library*

|  | String | Results |
| --- | --- | --- |
| #1 | (("we searched"):ab AND (review:ti,ab)) OR (rat OR rats OR mouse OR mice OR swine OR porcine OR murine OR sheep OR lambs OR pigs OR piglets OR rabbit OR rabbits OR cat OR cats OR dog OR dogs OR cattle OR bovine OR monkey OR monkeys OR trout OR marmoset* OR "animal experiment" OR "update review"):ti,ab,kw | 36,280 |
| #2 | MeSH descriptor: [Animal Experimentation] explode all trees | 11 |
| #3 | #1 OR #2 | 36,281 |
| #4 | (groups OR trial OR randomly OR placebo OR randomized OR randomised OR "controlled clinical trial" OR "randomized controlled trial"):ti,ab,kw | 1,608,423 |
| #5 | MeSH descriptor: [Controlled Clinical Trial] explode all trees | 40 |
| #6 | MeSH descriptor: [Randomized Controlled Trial] explode all trees | 37 |
| #7 | #4 OR #5 OR #6 | 1,608,423 |
| #8 | #7 NOT #3 | 1,577,265 |
| #9 | (diabetes OR diabetic OR diabets OR diabete OR "glucose intolerance" OR hyperglycemia OR hyperglucemia OR hyperglycaemia OR "hyper glycemia" OR "hyper glycaemia" OR hyperglycaemias OR hyperglycemias OR "hyperglycemic syndrome"):ti,ab,kw | 127,475 |
| #10 | MeSH descriptor: [Hyperglycemia] explode all trees | 4,003 |
| #11 | MeSH descriptor: [Diabetes Mellitus] explode all trees | 46,225 |
| #12 | #9 OR #10 OR #11 | 127,746 |
| #13 | (exercise OR "exercise training" OR "fitness training" OR "fitness workout" OR "physical exercise" OR exercises OR "physical activity" OR "physical activities" OR diet OR "dieting" OR "body weight" OR "weight decrease" OR "weight losing" OR "weight loss" OR "weight reducing" OR "weight reduction" OR "weight watching" OR "weight losses" OR "weight reductions" OR lifestyle OR lifestyles OR "life style" OR "life styles"):ti,ab,kw | 287,417 |
| #14 | MeSH descriptor: [Exercise] explode all trees | 39,051 |
| #15 | #13 OR #14 | 291,068 |
| #16 | (prevention OR intervention OR interventions OR interventional OR treatment OR treatments):ti,ab,kw | 1,417,194 |
| #17 | MeSH descriptor: [Diabetes, Gestational] explode all trees | 1,729 |
| #18 | ("gestational diabetes" OR "diabetes mellitus gravidarum" OR "diabetes mellitus of pregnancy" OR "diabetes, gestational" OR "diabetes, pregnancy" OR "diabetes of pregnancy" OR "pregnancy diabetes" OR "pregnancy-induced diabetes" OR GDM):ti,ab,kw | 4,194 |
| #19 | #17 OR #18 | 4,261 |
| #20 | #8 AND #12 AND #15 AND #16 AND #19 | 1,470 |

*Database: Embase*

|  | String | Results |
| --- | --- | --- |
| #1 | 'randomized controlled trial'/exp OR 'controlled clinical trial'/de OR random*:ti,ab,tt OR 'randomization'/de OR 'intermethod comparison'/de OR placebo:ti,ab,tt OR compare:ti,tt OR compared:ti,tt OR comparison:ti,tt OR ((evaluated:ab OR evaluate:ab OR evaluating:ab OR assessed:ab OR assess:ab) AND (compare:ab OR compared:ab OR comparing:ab OR comparison:ab)) OR ((open NEXT/1 label):ti,ab,tt) OR (((double OR single OR doubly OR singly) NEXT/1 (blind OR blinded OR blindly)):ti,ab,tt) OR 'double blind procedure'/de OR ((parallel NEXT/1 group*):ti,ab,tt) OR crossover:ti,ab,tt OR 'cross over':ti,ab,tt OR (((assign* OR match OR matched OR allocation) NEAR/6 (alternate OR group OR groups OR intervention OR interventions OR patient OR patients OR subject OR subjects OR participant OR participants)):ti,ab,tt) OR assigned:ti,ab,tt OR allocated:ti,ab,tt OR ((controlled NEAR/8 (study OR design OR trial)):ti,ab,tt) OR volunteer:ti,ab,tt OR volunteers:ti,ab,tt OR 'human experiment'/de OR trial:ti,tt | 6,818,239 |
| #2 | (((random* NEXT/1 sampl* NEAR/8 ('cross section*' OR questionnaire* OR survey OR surveys OR database OR databases)):ti,ab,tt) NOT ('comparative study'/de OR 'controlled study'/de OR 'randomised controlled':ti,ab,tt OR 'randomized controlled':ti,ab,tt OR 'randomly assigned':ti,ab,tt) OR ('cross-sectional study'/de NOT ('randomized controlled trial'/exp OR 'controlled clinical study'/de OR 'controlled study'/de OR 'randomised controlled':ti,ab,tt OR 'randomized controlled':ti,ab,tt OR 'control group':ti,ab,tt OR 'control groups':ti,ab,tt)) OR ('case control*':ti,ab,tt AND random*:ti,ab,tt NOT ('randomised controlled':ti,ab,tt OR 'randomized controlled':ti,ab,tt)) OR ('systematic review':ti,tt NOT (trial:ti,tt OR study:ti,tt)) OR (nonrandom*:ti,ab,tt NOT random*:ti,ab,tt) OR 'random field*':ti,ab,tt OR (('random cluster' NEAR/4 sampl*):ti,ab,tt) OR (review:ab AND review:it)) NOT trial:ti,tt OR ('we searched':ab AND (review:ti,tt OR review:it)) OR 'update review':ab OR ((databases NEAR/5 searched):ab) OR ((rat:ti,tt OR rats:ti,tt OR mouse:ti,tt OR mice:ti,tt OR swine:ti,tt OR porcine:ti,tt OR murine:ti,tt OR sheep:ti,tt OR lambs:ti,tt OR pigs:ti,tt OR piglets:ti,tt OR rabbit:ti,tt OR rabbits:ti,tt OR cat:ti,tt OR cats:ti,tt OR dog:ti,tt OR dogs:ti,tt OR cattle:ti,tt OR bovine:ti,tt OR monkey:ti,tt OR monkeys:ti,tt OR trout:ti,tt OR marmoset*:ti,tt) AND 'animal experiment'/de) OR ('animal experiment'/de NOT ('human experiment'/de OR 'human'/de)) | 4,569,164 |
| #3 | #1 NOT #2 | 5,998,112 |
| #4 | 'gestational diabetes'/exp OR 'diabetes mellitus gravidarum':ti,ab,kw OR 'diabetes mellitus of pregnancy':ti,ab,kw OR 'diabetes of pregnancy':ti,ab,kw OR 'diabetes, gestational':ti,ab,kw OR 'diabetes, pregnancy':ti,ab,kw OR 'gestational diabetes':ti,ab,kw OR 'pregnancy diabetes':ti,ab,kw OR 'pregnancy-induced diabetes':ti,ab,kw OR gdm:ti,ab,kw | 60,178 |
| #5 | 'prevention'/exp OR 'prevention':ti,ab,kw OR intervention:ti,ab,kw OR interventions:ti,ab,kw OR interventional:ti,ab,kw OR treatment:ti,ab,kw OR treatments:ti,ab,kw | 11,172,421 |
| #6 | 'exercise'/exp OR 'exercise':ti,ab,kw OR 'exercise training':ti,ab,kw OR 'fitness training':ti,ab,kw OR 'fitness workout':ti,ab,kw OR 'physical exercise':ti,ab,kw OR exercises:ti,ab,kw OR 'physical activity':ti,ab,kw OR 'physical activities':ti,ab,kw OR 'diet'/exp OR 'dieting':ti,ab,kw OR diet:ti,ab,kw OR 'body weight'/exp OR 'body weight':ti,ab,kw OR 'body weight loss'/exp OR 'weight decrease':ti,ab,kw OR 'weight losing':ti,ab,kw OR 'weight loss':ti,ab,kw OR 'weight reducing':ti,ab,kw OR 'weight reduction':ti,ab,kw OR 'weight watching':ti,ab,kw OR 'weight losses':ti,ab,kw OR 'weight reductions':ti,ab,kw OR 'lifestyle'/exp OR 'life style':ti,ab,kw OR 'lifestyle':ti,ab,kw OR 'life styles':ti,ab,kw OR lifestyles:ti,ab,kw | 2,501,082 |
| #7 | 'diabetes mellitus'/exp OR 'diabetes mellitus':ti,ab,kw,de OR 'diabetic':ti,ab,kw OR 'diabets':ti,ab,kw OR 'diabetes':ti,ab,kw OR 'diabete':ti,ab,kw OR 'glucose intolerance':ti,ab,kw OR 'glucose intolerance'/exp OR 'hyperglycemia'/exp OR 'glucose blood level, elevated':ti,ab,kw OR 'glycemia, hyper':ti,ab,kw OR 'high blood glucose':ti,ab,kw OR 'hyper-glycaemia':ti,ab,kw OR 'hyper-glycemia':ti,ab,kw OR 'hyperglucemia':ti,ab,kw OR 'hyperglycaemia':ti,ab,kw OR 'hyperglycaemias':ti,ab,kw OR 'hyperglycemia':ti,ab,kw OR 'hyperglycemias':ti,ab,kw OR 'hyperglycemic syndrome':ti,ab,kw | 1,635,481 |
| #8 | #3 AND #4 AND #5 AND #6 AND #7 | 3,018 |

*Database: Pubmed*

|  | String | Results |
| --- | --- | --- |
| #1 | "diabetes, gestational"[MeSH Terms] OR "gestational diabetes"[Title/Abstract] OR "Pregnancy-Induced Diabetes"[Title/Abstract] OR "GDM"[Title/Abstract] | 28,319 |
| #2 | "diabetes mellitus"[MeSH Terms] OR "diabetes"[Title/Abstract] OR "diabete"[Title/Abstract] OR "glucose intolerance"[Title/Abstract] OR "glucose intolerance"[MeSH Terms] OR "hyperglycemia"[MeSH Terms] OR "hyperglycaemia"[Title/Abstract] OR "hyperglycemia"[Title/Abstract] OR "hyperglycaemias"[Title/Abstract] OR "hyperglycemias"[Title/Abstract] | 868,752 |
| #3 | "Exercise"[MeSH Terms] OR "Exercise"[Title/Abstract] OR "Exercises"[Title/Abstract] OR "Physical Activity"[Title/Abstract] OR "Physical Activities"[Title/Abstract] OR "Diet"[MeSH Terms] OR "Diet"[Title/Abstract] OR "body weight"[MeSH Terms] OR "body weight"[Title/Abstract] OR "Weight Loss"[MeSH Terms] OR "Weight Loss"[Title/Abstract] OR "Weight Losses"[Title/Abstract] OR "Weight Reduction"[Title/Abstract] OR "Weight Reductions"[Title/Abstract] OR "Life Style"[MeSH Terms] OR "Life Style"[Title/Abstract] OR "Life Styles"[Title/Abstract] OR "Lifestyle"[Title/Abstract] OR "Lifestyles"[Title/Abstract] | 1,841,269 |
| #4 | "randomized controlled trial"[Publication Type] OR "controlled clinical trial"[Publication Type] OR "randomized"[Title/Abstract] OR "randomised"[Title/Abstract] OR "placebo"[Title/Abstract] OR "randomized"[Title/Abstract] OR "drug therapy"[MeSH Subheading] OR "random*"[Title/Abstract] OR "trial*"[Title/Abstract] OR "group*"[Title/Abstract] NOT ("animals"[MeSH Terms] NOT "humans"[MeSH Terms]) | 7,257,559 |
| #5 | "prevention"[Title/Abstract] OR "intervention"[Title/Abstract] OR "interventions"[Title/Abstract] OR "interventional"[Title/Abstract] OR "treatment"[Title/Abstract] OR "treatments"[Title/Abstract] | 7,233,483 |
| #6 | #1 AND #2 AND #3 AND #4 AND #5 | 2,230 |

*Database: Web of Science*

|  | String | Results |
| --- | --- | --- |
| #1 | TS=(("we searched" AND review) OR (rat OR rats OR mouse OR mice OR swine OR porcine OR murine OR sheep OR lambs OR pigs OR piglets OR rabbit OR rabbits OR cat OR cats OR dog OR dogs OR cattle OR bovine OR monkey OR monkeys OR trout OR marmoset* OR "animal experiment" OR "update review")) | 9,413,930 |
| #2 | TS=(groups OR trial OR randomly OR placebo OR randomized OR randomised OR "controlled clinical trial" OR "randomized controlled trial") | 15,417,807 |
| #3 | #2 NOT #1 | 13,909,876 |
| #4 | TS=(prevention OR intervention OR interventions OR interventional OR treatment OR treatments) | 17,007,819 |
| #5 | TS=("gestational diabetes" OR "diabetes mellitus gravidarum" OR "diabetes mellitus of pregnancy" OR "diabetes, gestational" OR "diabetes, pregnancy" OR "diabetes of pregnancy" OR "pregnancy diabetes" OR "pregnancy-induced diabetes" OR GDM) | 42,777 |
| #6 | TS=(exercise OR "exercise training" OR "fitness training" OR "fitness workout" OR "physical exercise" OR exercises OR "physical activity" OR "physical activities" OR diet OR "dieting" OR "body weight" OR "weight decrease" OR "weight losing" OR "weight loss" OR "weight reducing" OR "weight reduction" OR "weight watching" OR "weight losses" OR "weight reductions" OR lifestyle OR lifestyles OR "life style" OR "life styles") | 3,373,038 |
| #7 | TS=(diabetes OR diabetic OR diabets OR diabete OR "glucose intolerance" OR hyperglycemia OR hyperglucemia OR hyperglycaemia OR "hyper glycemia" OR "hyper glycaemia" OR hyperglycaemias OR hyperglycemias OR "hyperglycemic syndrome") | 1,567,236 |
| #8 | #3 AND #4 AND #5 AND #6 AND #7 | 2,694 |

# Supplementary Table 2. Excluded references after full text screening.

|  | Reference | Reason |
| --- | --- | --- |
| 1 | Abbas M, Giroux I, Donovan L, Mottola M, Miller D, Mcmanus RM. Diabetes-protective knowledge and behavior before and after the families defeating diabetes (FDD) intervention for women with recent GDM. Diabetes. 2017;66:A394. | Conference paper |
| 2 | Barton K, Donovan L, Giroux I, Miller D, Mottola M, Rosas-Arellano P, et al. Families defeating diabetes (FDD): Hemoglobin A1C testing at 3 and 12 months. Diabetes. 2016;65:A358. | Conference paper |
| 3 | Barton K, Donovan L, Giroux I, Miller D, Mottola M, McManus R. Glycated hemoglobin measurements at three, 12 and 24 months postpartum after gestational diabetes. Clin Invest Med. 2019;42(4):E37–41. | Same study |
| 4 | Beeharry DB, Swarbrick HS, Ahmed HA, Nair SN, Pond JP, Joseph FJ. A multimedia approach to diabetes structured education: The effects of “Keeping Healthy After Gestational Diabetes (GDM)”, a multimedia based education program on patient knowledge, self-efficacy and acceptability. Diabet Med. 2017;34:127. | No diabetes |
| 5 | Berks D, Hoedjes M, Franx A, Habbema D, Raat H, Duvekot H, et al. Postpartum lifestyle intervention after complicated pregnancy proves feasible. Pregnancy Hypertens. 2010;1:S25. | Wrong study design |
| 6 | Berks D, Hoedjes M, Raat H, Franx A, Looman CWN, Van Oostwaard MF, et al. Feasibility and effectiveness of a lifestyle intervention after complicated pregnancies to improve risk factors for future cardiometabolic disease. Pregnancy Hypertens. 2019;15:98–107. | Wrong population |
| 7 | Brazeau AS, Leong A, Meltzer SJ, Cruz R, DaCosta D, Hendrickson-Nelson M, et al. Group-based activities with on-site childcare and online support improve glucose tolerance in women within 5 years of gestational diabetes pregnancy. Cardiovasc Diabetol. 2014;13:104. | Wrong study design |
| 8 | Brown SD, Guterman J, Gordon N, Tsai AL, Hedderson MM, Ferrara A. Evaluating a postpartum diabetes prevention program: The gestational diabetes’ effects on moms (GEM) trial. Diabetes. 2017;66:A216. | Conference paper |
| 9 | Brown SD, Hedderson MM, Gordon N, Albright CL, Tsai AL, Quesenberry CP, et al. Reach, acceptability, and perceived success of a telehealth diabetes prevention program among racially and ethnically diverse patients with gestational diabetes: the GEM cluster-randomized trial. Transl Behav Med. 2022;12(7):793–9. | Same study |
| 10 | Cheung NW, Simmons D, Marschner S, Thiagalingam A, Pasupathy D, Smith BJ, et al. Randomised controlled trial of a customised text messaging and activity monitor program for lifestyle improvement after gestational diabetes. Nutrients. 2024;16(6):820. | No diabetes |
| 11 | Ferrara A, Ehrlich SF, Feng J, Quesenberry CP, Moore SD, Hedderson MM. Postpartum weight loss is associated with improved glucose and insulin homeostasis in women with a history of gestational diabetes (GDM). Diabetes. 2012;61:A339. | Conference paper |
| 12 | Ferrara A, Hedderson MM, Albright CL, Brown SD, Ehrlich SF, Mevi AA, et al. Reduced postpartum weight retention with a DPP-derived lifestyle intervention: The gestational diabetes’ effects on MOMS (GEM) cluster randomized trial. Diabetes. 2014;63:A94–5. | Same study |
| 13 | Guo J, Long Q, Yang J, Lin Q, Wiley J, Chen JL. The efficacy of an intensive lifestyle modification program on psychosocial outcomes among rural women with prior gestational diabetes mellitus: six months follow-up of a randomized controlled trial. Int J Environ Res Public Health. 2021;18(4):1519. | No diabetes |
| 14 | Hannon TS, Saha CK, Carroll AE, Palmer KNB, Phillips EO, Marrero DG. The ENCOURAGE healthy families study: A comparative effectiveness trial to reduce risk for type 2 diabetes in mothers and children. Pediatr Diabetes. 2018;19(6):1041–9. | No diabetes |
| 15 | Hedderson MM, Brown SD, Feng J, Quesenberry C, Ferrara A. A translational diabetes prevention program (DPP)-based lifestyle intervention in women with GDM improves postpartum lifestyle behaviors. Diabetes. 2019;68(Suppl 1):1425-P. | Same study |
| 16 | Holmes VA, Draffin CR, Patterson CC, Francis L, Irwin J, McConnell M, et al. Postnatal lifestyle intervention for overweight women with previous gestational diabetes: a randomized controlled trial. J Clin Endocrinol Metab. 2018;103(7):2478–87. | No diabetes |
| 17 | Horwitz MEM, Edwards CV, Athavale P, McCloskey L, Cabral HJ, Benjamin EJ, et al. The STAR-MAMA RCT: bilingual mobile health coaching for postpartum weight loss. Am J Prev Med. 2023;65(4):596–607. | No diabetes |
| 18 | Hu G, Tian H, Zhang F, Liu H, Zhang C, Zhang S, et al. Tianjin gestational diabetes mellitus prevention program: Study design, methods, and 1-year interim report on the feasibility of lifestyle intervention program. Diabetes. 2012;61:A39. | Conference paper |
| 19 | Huvinen E, Koivusalo SB, Meinilä J, Valkama A, Tiitinen A, Rönö K, et al. Effects of a lifestyle intervention during pregnancy and first postpartum year: findings from the RADIEL study. J Clin Endocrinol Metab. 2018;103(4):1669–77. | Wrong population |
| 20 | Jelsma JGM, van Poppel MNM, Smith BJ, Cinnadaio N, Bauman A, Tapsell L, et al. Changing psychosocial determinants of physical activity and diet in women with a history of gestational diabetes mellitus. Diabetes Metab Res Rev. 2018;34(1):e2942. | No diabetes |
| 21 | Kapoor D, Gupta Y, Desai A, Praveen D, Joshi R, Rozati R, et al. Lifestyle intervention programme for Indian women with history of gestational diabetes mellitus. Glob Health Epidemiol Genom. 2019;4:e1. | Same study |
| 22 | Kim C, Draska M, Hess ML, Wilson EJ, Richardson CR. A web-based pedometer programme in women with a recent history of gestational diabetes. Diabet Med. 2012;29(2):278–83. | No diabetes |
| 23 | Kim SH, Kim HJ, Shin G. Self-management mobile virtual reality program for women with gestational diabetes. Int J Environ Res Public Health. 2021;18(4):1539. | Wrong population |
| 24 | Li M, Lin Q, Shi J, Xi Y, Xiang C, Yong C, et al. The impact of lifestyle intervention on dietary quality among rural women with previous gestational diabetes mellitus-a randomized controlled study. Nutrients. 2021;13(8):2642. | No diabetes |
| 25 | Liew SJ, Soon CS, Chooi YC, Tint MT, Eriksson JG. A holistic approach to preventing type 2 diabetes in Asian women with a history of gestational diabetes mellitus: a feasibility study and pilot randomized controlled trial. Front Clin Diabetes Healthc. 2023;4:1251411. | Wrong study duration |
| 26 | Lim K, Chan SY, Lim SL, Tai BC, Tsai C, Wong SR, et al. A smartphone app to restore optimal weight (SPAROW) in women with recent gestational diabetes mellitus: randomized controlled trial. JMIR Mhealth Uhealth. 2021;9(3):e22147. | No diabetes |
| 27 | Lim S, Versace VL, O’Reilly S, Janus E, Dunbar J. Weight change and cardiometabolic outcomes in postpartum women with history of gestational diabetes. Nutrients. 2019;11(4):922. | Same study |
| 28 | Lipscombe LL, Delos-Reyes F, Glenn AJ, de Sequeira S, Liang X, Grant S, et al. The avoiding diabetes after pregnancy trial in moms program: feasibility of a diabetes prevention program for women with recent gestational diabetes mellitus. Can J Diabetes. 2019;43(8):613–20. | No diabetes |
| 29 | Liu H, Wang L, Zhang S, Leng J, Li N, Li W, et al. 1 year weight losses in the Tianjin gestational diabetes mellitus prevention program: a randomised trial. Lancet Diabetes Endocrinol. 2016;4:S11. | Conference paper |
| 30 | Liu H, Wang L, Zhang S, Leng J, Li N, Li W, et al. One-year weight losses in the Tianjin gestational diabetes mellitus prevention programme: a randomized clinical trial. Diabetes Obes Metab. 2018;20(5):1246–55. | Same study |
| 31 | McCance DR, Draffin C, Patterson CC, Francis L, Irwin J, McConnell M, et al. Postnatal lifestyle intervention for overweight women with previous gestational diabetes mellitus (PAIGE): A pilot randomised controlled trial. Ir J Med Sci. 2016;185(7):S401. | Conference paper |
| 32 | McIntyre HD, Peacock A, Miller YD, Koh D, Marshall AL. Pilot study of an individualised early postpartum intervention to increase physical activity in women with previous gestational diabetes. Int J Endocrinol. 2012;2012:892019. | No diabetes |
| 33 | McManus RM, Donovan L, Miller D, Mottola M, Giroux I, Rosas-Arellano P. Families defeating diabetes (FDD): A Canadian intervention for family-centered diabetes prevention following gestational diabetes (GDM): Initial results. Diabetes. 2015;64:A632. | Conference paper |
| 34 | Nicklas JM, Leiferman J, Pyle L, Soares A, Bull S, Tong S, et al. Feasibility of an mhealth postpartum lifestyle intervention for women with cardiometabolic risk pre-and mid-covid: The fit after baby pilot randomized controlled trial. J Gen Intern Med. 2021;36:S178. | Wrong population |
| 35 | Nicklas JM, Zera CA, Levkoff SE, Seely EW. Effect of postpartum weight change on risk factors for type 2 diabetes among women with recent gestational diabetes. Diabetes. 2015;64:A375. | Same study |
| 36 | Nicklas JM, Zera CA, Rosner BA, Levkoff SE, Seely EW. A web-based lifestyle intervention to decrease postpartumweight retention inwomenwith recent gestational diabetes mellitus: The balance after baby pilot RCT. J Gen Intern Med. 2013;28:S14. | Conference paper |
| 37 | Nicklas JM, Rosner BA, Zera CA, Seely EW. Association between changes in postpartum weight and waist circumference and changes in cardiometabolic risk factors among women with recent gestational diabetes. Prev Chronic Dis. 2019;16:E47. | Same study |
| 38 | Nicklas JM, Skurnik G, Roche AT, Schultz C, Suresh K, Seely EW. A web-based lifestyle intervention to reduce postpartum weight retention in women with recent gestational diabetes: the balance after baby intervention trial. Diabetes. 2020;69(Suppl 1):191-OR. | Same study |
| 39 | O’Dea A, Tierney M, McGuire B, Newell JN, Glynn L, Gibson I, et al. An evaluation of Croí MyAction community lifestyle modification programme compared to standard care to reduce progression to diabetes/prediabetes in women with prior gestational diabetes mellitus (GDM). Ir J Med Sci. 2014;183(9):S461. | Conference paper |
| 40 | O’Dea A, Tierney M, McGuire BE, Newell J, Glynn LG, Gibson I, et al. Can the onset of type 2 diabetes be delayed by a group-based lifestyle intervention in women with prediabetes following gestational diabetes mellitus (GDM)? Findings from a randomized control mixed methods trial. J Diabetes Res. 2015;2015:798460. | No diabetes |
| 41 | O’Reilly SL, Dunbar JA, Versace V, Janus E, Best JD, Carter R, et al. Mothers after gestational diabetes in australia (MAGDA): a randomised controlled trial of a postnatal diabetes prevention program. PLoS Med. 2016;13(7):e1002092. | No diabetes |
| 42 | Palnati M, Marcus BH, Pekow P, Rosal MC, Manson JE, Chasan-Taber L. The impact of a lifestyle intervention on postpartum weight retention among at-risk hispanic women. Am J Prev Med. 2021;61(1):44–54. | No diabetes |
| 43 | Parsons J. Development and feasibility study of a lifestyle intervention to reduce the risk of Type 2 diabetes for women with recent gestational diabetes : the GODDESS study [Thesis (Doctor of Philosophy)]. [London]: Florence Nightingale Faculty of Nursing, Midwifery and Palliative Care King’s College; 2022. | Same study |
| 44 | Peacock AS, Bogossian FE, Wilkinson SA, Gibbons KS, Kim C, McIntyre HD. A randomised controlled trial to delay or prevent type 2 diabetes after gestational diabetes: walking for exercise and nutrition to prevent diabetes for you. Int J Endocrinol. 2015;2015:423717. | No diabetes |
| 45 | Ratner RE, Christophi CA, Metzger BE, Dabelea D, Bennett PH, Pi-Sunyer X, et al. Prevention of diabetes in women with a history of gestational diabetes: effects of metformin and lifestyle interventions. J Clin Endocrinol Metab. 2008;93(12):4774–9. | Wrong population |
| 46 | Reinhardt JA, van der Ploeg HP, Grzegrzulka R, Timperley JG. lmplementing lifestyle change through phone-based motivational interviewing in rural-based women with previous gestational diabetes mellitus. Health Promot J Austr. 2012;23(1):5–9. | No diabetes |
| 47 | Reutrakul S, Martyn-Nemeth P, Quinn L, Danielson K, Rydzon B, Baron K, et al. Effects of sleep-extend on glucose metabolism in women with a history of gestational diabetes: A pilot study. Sleep. 2021;44:A261. | Conference paper |
| 48 | Reutrakul S, Martyn-Nemeth P, Quinn L, Rydzon B, Priyadarshini M, Danielson KK, et al. Effects of Sleep-Extend on glucose metabolism in women with a history of gestational diabetes: a pilot randomized trial. Pilot Feasibility Stud. 2022;8(1):119. | No diabetes |
| 49 | Rollo ME, Baldwin JN, Hutchesson M, Aguiar EJ, Wynne K, Young A, et al. The feasibility and preliminary efficacy of an eHealth lifestyle program in women with recent gestational diabetes mellitus: a pilot study. Int J Environ Res Public Health. 2020;17(19):7115. | No diabetes |
| 50 | Sangeetha-Shyam, Fatimah A, Rohana A, Norasyikin A, Karuthan C, Nik S, et al. Lowering dietary glycaemic index through nutrition education among Malaysian women with a history of gestational diabetes mellitus. Malays J Nutr. 2013;19(1):9–23. | Same study |
| 51 | Shyam S, Arshad F, Nisak MYB, Safie NS, Kamaruddin NA, Abdul Ghani R, et al. Effect of low gi diet vs low fat diet on metabolic risk markers in women post gestational diabetes mellitus (PGDM): A preliminary finding. J Diabetes. 2011;3:87. | Conference paper |
| 52 | Smith BJ, Cinnadaio N, Cheung NW, Bauman A, Tapsell LC, van der Ploeg HP. Investigation of a lifestyle change strategy for high-risk women with a history of gestational diabetes. Diabetes Res Clin Pract. 2014;106(3):e60-63. | No diabetes |
| 53 | Tandon N, Kapoor D, Lakshmi JK, Bhattacharya A, Billot L, Patel A. A lifestyle intervention to prevent deterioration of glycaemic status among women with previous gestational diabetes: The LIVING trial. Diabetologia. 2021;64:S145. | Conference paper |
| 54 | Taylor R, Rollo ME, Baldwin JN, Hutchesson M, Aguiar EJ, Wynne K, et al. Evaluation of a Type 2 diabetes risk reduction online program for women with recent gestational diabetes: a randomised trial. Int J Behav Nutr Phys Act. 2022;19(1):35. | No diabetes |
| 55 | Tsoi KY, Chan RCM, Zhang C, Tam WH, Ma RCW. A randomized controlled trial to evaluate the effects of an early postnatal lifestyle modification program on diet, adiposity and metabolic outcome in mothers with gestational diabetes mellitus. Int J Gynaecol Obstet. 2024;166(3):1170–82. | No diabetes |
| 56 | Wagner K, Prakash V, St Laurent C, Marcus B, Pekow P, Rosal M, et al. The impact of a lifestyle intervention on pregnancy and postpartum cardiovascular and insulin resistance biomarkers among at-risk latinas. Paediatr Perinat Epidemiol. 2021;35:43. | Conference paper |
| 57 | Wagner KA, St Laurent CW, Pekow P, Marcus B, Rosal MC, Braun B, et al. The impact of a lifestyle intervention on postpartum cardiometabolic risk factors among hispanic women with abnormal glucose tolerance during pregnancy: secondary analysis of a randomized trial. J Phys Act Health. 2024;21(1):40–50. | Same study |
| 58 | Zhong Q, Chen Y, Luo M, Lin Q, Tan J, Xiao S, et al. The 18-month efficacy of an Intensive LifeStyle Modification Program (ILSM) to reduce type 2 diabetes risk among rural women: a cluster randomized controlled trial. Global Health. 2023;19(1):6. | No diabetes |

# Supplementary Table 3. Risk of bias assessment for the incidence of diabetes in women with recent gestational diabetes.

| **Study** | **Bias arising from the randomization process** | | **Bias due to deviations from intended interventions** | | **Bias due to missing outcome data** | | **Bias in measurement of the outcome** | | **Bias in selection of the reported result** | | **Overall bias** | |
| --- | --- | --- | --- | --- | --- | --- | --- | --- | --- | --- | --- | --- |
|  | Rank | Comment | Rank | Comment | Rank | Comment | Rank | Comment | Rank | Comment | Rank | Comment |
| Wein et al., 1999 | SC | Randomization method not informed. Baseline differences look compatible with chance. | Low | Open label trial. Some deviations but we have no reason to believe that they were due to the trial context. ITT analysis. | Low | Incomplete follow-up (96.5%), but losses were less frequent than cases and similar in both groups. | Low | Outcome based on laboratory measurements, unlikely to be influenced by knowledge of the intervention received. | Low | Probably analyzed as planned. | Low | Even though the algorithm’s overall assessment indicated some concerns (SC), we judged this study (n=200), with long follow-up and many events, as having overall high quality |
| Cheung et al., 2011 | SC | Randomization method not informed. Minimal baseline differences. | SC | Open label trial. Some deviations but we have no reason to believe they were due to the trial context. Incident diabetes not a listed endpoint for analysis. | SC | Incomplete follow-up (74.4%). Few events with more (8x) losses than cases. | Low | Outcome based on laboratory measurements is unlikely to be influenced by knowledge of the intervention received. | Low | Diabetes incidence not analyzed. | SC |  |
| Ji et al., 2011 | SC | Randomization method not informed. No baseline differences are apparent. | Low | Open label trial. Possible deviations but we have no reason to believe they were due to the trial context. | SC | Incomplete follow-up (90.3%). Few events with more (4.7x) losses than cases. | Low | Outcome based on laboratory measurements is unlikely to be influenced by knowledge of the intervention received. | Low | Diabetes not specified as primary outcome. | SC |  |
| Yu et al., 2012 | SC | Randomization method not informed. No baseline differences are apparent. | Low | Open label trial. Possible deviations but we have no reason to believe they were due to the trial context. | Low | Incomplete follow-up (94.0%), but losses were less frequent than cases and similar in both groups. | Low | Outcome based on laboratory measurements is unlikely to be influenced by knowledge of the intervention received. | Low | Diabetes not specified as primary outcome. | SC |  |
| Shyam et al., 2013 | Low | Randomization by computer generated sequence. No baseline differences are apparent. | Low | Open label trial. Possible deviations but we have no reason to believe they were due to the trial context. | SC | Incomplete follow-up. (84.4%). Few events with more (4.0x) losses than cases. | Low | Outcome based on laboratory measurements is unlikely to be influenced by knowledge of the intervention received. | Low | Diabetes not specified as primary outcome. | SC |  |
| Guo et al., 2013 | SC | Randomization by random number table. No statistical differences in baseline characteristics reported in this small study. | Low | Open label trial. Possible deviations but we have no reason to believe they were due to the trial context. | Low | Apparently complete follow-up. | Low | Outcome based on laboratory measurements is unlikely to be influenced by knowledge of the intervention received. | Low | Probably analyzed as planned. | SC |  |
| Geng et al., 2014 | High | Historic controls. Some baseline differences are apparent. No group comparison for baseline characteristics. | High | Comparison with historic controls producing deviations due to the trial context. | Low | Apparently complete follow-up. | Low | Outcome based on laboratory measurements is unlikely to be influenced by knowledge of the intervention received. | Low | Probably analyzed as planned. | High |  |
| Nicklas et al., 2014 | Low | Computer generated sequence, with allocation using sealed envelopes. Baseline differences look compatible with chance. | Low | Open label trial. Possible deviations but we have no reason to believe they were due to the trial context. | SC | Incomplete follow-up. (84.0%). Few events with more (4.0x) losses than cases. | Low | Outcome based on laboratory measurements is unlikely to be influenced by knowledge of the intervention received. | Low | Diabetes not specified as primary outcome. | SC |  |
| Shek et al., 2014 | Low | Computer generated randomization sequence. Baseline differences look compatible with chance. | Low | Open label trial. Possible deviations but we have no reason to believe they were due to the trial context. ITT analysis. | Low | Incomplete follow-up (94.0%), but losses were less frequent than cases and similar in both groups. | Low | Outcome based on laboratory measurements is unlikely to be influenced by knowledge of the intervention received. | Low | Probably analyzed as planned. | Low |  |
| Pérez-Ferre et al., 2015 | SC | Randomization method not informed. Baseline differences look compatible with chance. | Low | Open label trial. Possible deviations but we have no reason to believe they were due to the trial context. ITT analysis. | Low | Incomplete follow-up (93.5%), but losses were less frequent than cases. | Low | Outcome based on laboratory measurements is unlikely to be influenced by knowledge of the intervention received. | Low | Probably analyzed as planned. | SC |  |
| Ferrara et al., 2016 | Low | Clinics randomly selected by computer generated scheme. No baseline differences are apparent. | Low | Blinded trial. Possible deviations but we have no reason to believe they were due to the trial context. ITT analysis. | Low | Incomplete follow-up (90.8%) with somewhat more losses than cases but similar in both groups. | Low | Outcome based on laboratory measurements is unlikely to be influenced by knowledge of the intervention received. | Low | Diabetes as an exploratory outcome. Study protocol published.^1^ | Low |  |
| Zilberman-Kravits et al., 2018 | High | Alternation used for treatment allocation. No baseline differences are apparent. | Low | Open label trial. Possible deviations but we have no reason to believe they were due to the trial context. | High | Incomplete follow-up (57.8%) in a study with no cases. | Low | Outcome based on laboratory measurements is unlikely to be influenced by knowledge of the intervention received. | Low | Diabetes not specified as primary outcome. | High |  |
| McManus et al., 2018 | Low | Web-based randomization system. Minimal baseline differences. | Low | Open label trial. Possible deviations but we have no reason to believe they were due to the trial context. | High | Incomplete follow-up (46.5%). Few events with many more (45.5x) losses than cases. | Low | Outcome based on laboratory measurements is unlikely to be influenced by knowledge of the intervention received. | Low | Diabetes not specified as primary outcome. | High |  |
| Cheung et al., 2019 | Low | Randomization scheme generated by computer. Baseline differences look compatible with chance. | Low | Open label trial. Possible deviations but we have no reason to believe they were due to the trial context. | High | Incomplete follow-up (38.3%). Few events with many more (18.5x) losses than cases | Low | Outcome based on laboratory measurements is unlikely to be influenced by knowledge of the intervention received. | Low | Diabetes not specified as primary outcome. | High |  |
| Hu et al., 2022 | Low | Randomization by computer generated scheme kept centrally. No baseline differences are apparent. | Low | Open label trial. Possible deviations but we have no reason to believe they were due to the trial context. ITT analysis. | SC | Not informed. | Low | Outcome based on laboratory measurements is unlikely to be influenced by knowledge of the intervention received. | Low | Probably analyzed as planned. Study protocol published.^2^ | SC |  |
| Tandon et al., 2022 | Low | Web-based randomization system. Baseline differences look compatible with chance. | Low | Open label trial. Some deviations but we have no reason to believe they were due to the trial context. ITT analysis. | Low | Incomplete follow-up (89.1%), with losses similar in both groups. | Low | Outcome based on laboratory measurements is unlikely to be influenced by knowledge of the intervention received. | Low | Probably analyzed as planned. Study protocol published.^3^ | Low |  |
| Potzel et al., 2022 | SC | Web-based randomization system. Baseline differences present in a small sample. | Low | Open label trial. Possible deviations but we have no reason to believe they were due to the trial context. | SC | Incomplete follow-up (86.4%). Few events with more (4.5x) losses than cases. | Low | Outcome based on laboratory measurements is unlikely to be influenced by knowledge of the intervention received. | Low | Diabetes not specified as primary outcome. | SC |  |
| Parsons et al., 2022 | Low | Computer generated sequence, with allocation using sealed envelopes. Baseline differences by initial allocation not informed. | High | Open label trial. Some deviations due to the trial context. | High | Incomplete follow-up (56.0%). Few events with many more (22.0x) losses than cases | Low | Outcome based on laboratory measurements is unlikely to be influenced by knowledge of the intervention received. | Low | Diabetes not specified as primary outcome. | High |  |
| Lee et al., 2022 | Low | Clinics randomly selected by computer generated scheme. Baseline differences look compatible with chance. | Low | Probable open label trial. Possible deviations but we have no reason to believe they were due to the trial context. ITT analysis. | SC | Incomplete follow-up (51.1%). More (5.2x) losses than cases. | Low | Outcome based on laboratory measurements is unlikely to be influenced by knowledge of the intervention received. | Low | Probably analyzed as planned. | SC |  |
| Quansah et al., 2023 | Low | Computer generated sequence, with allocation using sealed envelopes. Baseline differences look compatible with chance. | Low | Open label trial. Some deviations but we have no reason to believe they were due to the trial context. ITT analysis. | SC | Incomplete follow-up (87.9%). Few events with more (4.2x) losses than cases. | Low | Outcome based on laboratory measurements is unlikely to be influenced by knowledge of the intervention received. | Low | Diabetes not specified as primary outcome.  Study protocol published.^4^ | SC |  |
| Iqbal et al., 2024 | Low | Computer generated sequence, with allocation using sealed envelopes. Baseline differences look compatible with chance. | Low | Open label trial. Some deviations but we have no reason to believe they were due to the trial context. | Low | Incomplete follow-up (82.8%), with losses similar to cases and similar in both groups. | Low | Outcome based on laboratory measurements is unlikely to be influenced by knowledge of the intervention received. | Low | Diabetes not specified as primary outcome. | Low |  |
| Minschart et al., 2024 | Low | Randomization by computer generated sequence. Baseline differences look compatible with chance. | Low | Open label trial. Some deviations but we have no reason to believe they were due to the trial context. ITT analysis. | SC | Incomplete follow-up (78.3%). Few events with more (6.5x) losses than cases. | Low | Outcome based on laboratory measurements, unlikely to be influenced by knowledge of the intervention received. | Low | Diabetes not specified as primary outcome.  Study protocol published.^5^ | SC |  |
| Sundarapperuma et al., 2024 | High | Small number of clinics selected by random number table. Important baseline differences in a small sample suggest a problem with the randomization process. Mean baseline HbA1c of controls in the diabetes range. | Low | Probable open label trial. Possible deviations but we have no reason to believe they were due to the trial context. | SC | Incomplete follow-up (76.0), with losses similar in both groups. | Low | Outcome based on laboratory measurements, unlikely to be influenced by knowledge of the intervention received. | Low | Diabetes not specified as primary outcome. | High |  |
| Schmidt et al., 2024 | Low | Web-based randomization system. Baseline differences compatible with chance. | Low | Open label trial. Some deviations but we have no reason to believe they were due to the trial context. ITT analysis. | Low | Incomplete follow-up (90.8%), but losses were less frequent than cases and similar in both groups. | Low | Outcome based on laboratory measurements, unlikely to be influenced by knowledge of the intervention received. | Low | Probably analyzed as planned. Study protocol published.^6^ | Low |  |

# Supplementary Table 4. Risk of bias assessment for the weight change in women with recent gestational diabetes.

| **Study** | **Bias arising from the randomization process** | | **Bias due to deviations from intended interventions** | | **Bias due to missing outcome data** | | **Bias in measurement of the outcome** | | **Bias in selection of the reported result** | | **Overall bias** | |
| --- | --- | --- | --- | --- | --- | --- | --- | --- | --- | --- | --- | --- |
|  | Rank | Comment | Rank | Comment | Rank | Comment | Rank | Comment | Rank | Comment | Rank | Comment |
| Sheng et al., 2012 | SC | Randomization method not informed. No baseline differences are apparent. | Low | Open label trial. Possible deviations but we have no reason to believe they were due to the trial context. | Low | Incomplete follow-up (90.3%). | Low | Weight measured objectively, unlikely to be influenced by knowledge of the intervention received. | Low | Probably analyzed as planned. | SC |  |
| Shyam et al., 2013 | Low | Randomization by computer generated sequence. No baseline differences are apparent. | Low | Open label trial. Possible deviations but we have no reason to believe they were due to the trial context. | Low | Incomplete follow-up (84.4%). | Low | Weight measured objectively, unlikely to be influenced by knowledge of the intervention received. | Low | Probably analyzed as planned. | Low |  |
| Geng et al., 2014 | High | Historic controls. Some baseline differences are apparent. No group comparison for baseline characteristics. | High | Comparison with historic controls producing deviations due to the trial context. | Low | Apparently complete follow-up. | Low | Weight measured objectively, unlikely to be influenced by knowledge of the intervention received. | Low | Probably analyzed as planned. | High |  |
| Nickas et al., 2014 | Low | Computer generated sequence, with allocation using sealed envelopes. Baseline differences look compatible with chance. | Low | Open label trial. Possible deviations but we have no reason to believe they were due to the trial context. | Low | Incomplete follow-up (80.0%) | Low | Weight measured objectively, unlikely to be influenced by knowledge of the intervention received. | Low | Probably analyzed as planned. | Low |  |
| Ferrara et al., 2016 | Low | Clinics randomly selected by computer generated scheme. No baseline differences are apparent. | Low | Blinded trial. Possible deviations but we have no reason to believe they were due to the trial context. ITT analysis. | Low | Incomplete follow-up (90.8%). | Low | Weight measured objectively, unlikely to be influenced by knowledge of the intervention received. | Low | Probably analyzed as planned. Study protocol published.^1^ | Low |  |
| Zilberman-Kravits et al., 2018 | High | Alternation used for treatment allocation. No baseline differences are apparent. | Low | Open label trial. Possible deviations but we have no reason to believe they were due to the trial context. | SC | Incomplete follow-up (57.8%). | Low | Weight measured objectively, unlikely to be influenced by knowledge of the intervention received. | Low | Probably analyzed as planned. | High |  |
| McManus et al., 2018 | Low | Web-based randomization system. Minimal baseline differences. | Low | Open label trial. Possible deviations but we have no reason to believe they were due to the trial context. | High | Incomplete follow-up (46.5%). | Low | Weight measured objectively, unlikely to be influenced by knowledge of the intervention received. | Low | Probably analyzed as planned. | High |  |
| Cheung et al., 2019 | Low | Randomization scheme generated by computer. Baseline differences look compatible with chance. | Low | Open label trial. Possible deviations but we have no reason to believe they were due to the trial context. | High | Incomplete follow-up (38.3%). | High | Outcome was self-reported, possibly influenced by knowledge of the intervention received. | Low | Probably analyzed as planned. | High |  |
| Liu et al., 2018 | Low | Randomization by computer generated scheme kept centrally. No baseline differences are apparent. | Low | Open label trial. Possible deviations but we have no reason to believe they were due to the trial context. ITT analysis. | SC | Incomplete follow-up (78.8%). | Low | Weight measured objectively, unlikely to be influenced by knowledge of the intervention received. | Low | Probably analyzed as planned. Study protocol published.^2^ | SC |  |
| Tandon et al., 2022 | Low | Web-based randomization system. Baseline differences look compatible with chance. | Low | Open label trial. Some deviations but we have no reason to believe they were due to the trial context. ITT analysis. | Low | Incomplete follow-up (89.1%). | Low | Weight measured objectively, unlikely to be influenced by knowledge of the intervention received. | Low | Probably analyzed as planned. Study protocol published.^3^ | Low |  |
| Parsons et al., 2022 | Low | Computer generated sequence, with allocation using sealed envelopes. Baseline differences by initial allocation not informed. | High | Open label trial. Some deviations due to the trial context. | SC | Incomplete follow-up (56.0%). | Low | Weight measured objectively, unlikely to be influenced by knowledge of the intervention received. | Low | Probably analyzed as planned. | High |  |
| Lee et al., 2022 | Low | Clinics randomly selected by computer generated scheme. Baseline differences look compatible with chance. | Low | Probable open label trial. Possible deviations but we have no reason to believe they were due to the trial context. ITT analysis. | SC | Incomplete follow-up (51.1%). | Low | Weight measured objectively, unlikely to be influenced by knowledge of the intervention received. | Low | Probably analyzed as planned. | SC |  |
| Quansah et al., 2023 | Low | Computer generated sequence, with allocation using sealed envelopes. Baseline differences look compatible with chance. | Low | Open label trial. Some deviations but we have no reason to believe they were due to the trial context. ITT analysis. | Low | Incomplete follow-up (87.9%). | Low | Weight measured objectively, unlikely to be influenced by knowledge of the intervention received. | Low | Probably analyzed as planned. | Low |  |
| Iqbal et al., 2024 | Low | Computer generated sequence, with allocation using sealed envelopes. Baseline differences look compatible with chance. | Low | Open label trial. Some deviations but we have no reason to believe they were due to the trial context. | Low | Incomplete follow-up (82.8%). | Low | Weight measure objectively, unlikely to be influenced by knowledge of the intervention received. | Low | Probably analyzed as planned. | Low |  |
| Minschart et al., 2024 | Low | Randomization by computer generated sequence. Baseline differences look compatible with chance. | Low | Open label trial. Some deviations but we have no reason to believe they were due to the trial context. ITT analysis. | SC | Incomplete follow-up (78.3%). | Low | Weight measure objectively, unlikely to be influenced by knowledge of the intervention received. | Low | Probably analyzed as planned. Study protocol published.^5^ | SC |  |
| Schmidt et al., 2024 | Low | Web-based randomization system. Baseline differences compatible with chance. | Low | Open label trial. Some deviations but we have no reason to believe they were due to the trial context. ITT analysis. | Low | Incomplete follow-up (90.8%). | Low | Weight measure objectively, unlikely to be influenced by knowledge of the intervention received. | Low | Probably analyzed as planned. Study protocol published.^6^ | Low |  |

**References**

1. Ferrara A, Hedderson MM, Albright CL, Brown SD, Ehrlich SF, Caan BJ, et al. A pragmatic cluster randomized clinical trial of diabetes prevention strategies for women with gestational diabetes: design and rationale of the Gestational Diabetes’ Effects on Moms (GEM) study. BMC Pregnancy Childbirth. 2014 Jan 15;14(1):21.

2. Hu G, Tian H, Zhang F, Liu H, Zhang C, Zhang S, et al. Tianjin Gestational Diabetes Mellitus Prevention Program: Study design, methods, and 1-year interim report on the feasibility of lifestyle intervention program. Diabetes Res Clin Pract. 2012 Dec 1;98(3):508–17.

3. Gupta Y, Kapoor D, Josyula LK, Praveen D, Naheed A, Desai AK, et al. A lifestyle intervention programme for the prevention of Type 2 diabetes mellitus among South Asian women with gestational diabetes mellitus [LIVING study]: protocol for a randomized trial. Diabet Med. 2019;36(2):243–51.

4. Horsch A, Gilbert L, Lanzi S, Gross J, Kayser B, Vial Y, et al. Improving cardiometabolic and mental health in women with gestational diabetes mellitus and their offspring: study protocol for *MySweetHeart Trial* , a randomised controlled trial. BMJ Open. 2018 Feb;8(2):e020462.

5. Minschart C, Maes T, De Block C, Van Pottelbergh I, Myngheer N, Abrams P, et al. Mobile-Based Lifestyle Intervention in Women with Glucose Intolerance after Gestational Diabetes Mellitus (MELINDA), A Multicenter Randomized Controlled Trial: Methodology and Design. J Clin Med. 2020 Aug;9(8):2635.

6. Schmidt MI, Duncan BB, Castilhos C, Wendland EM, Hallal PC, Schaan BD, et al. Lifestyle INtervention for Diabetes prevention After pregnancy (LINDA-Brasil): study protocol for a multicenter randomized controlled trial. BMC Pregnancy Childbirth. 2016;16(1):68.

# Supplementary Table 5. Evaluation of the quality of the evidence using the GRADE system.

| **Outcome** | **No. of Studies**  **(Participants, events)** | **Study Design** | **Risk of Bias**  **(RoB-2)** | | **Inconsistency**  **(I²)** | **Indirectness** | **Imprecision** | **Publication Bias** | **Effect Size**  **(95% CI)** | **Certainty of Evidence (GRADE)** |
| --- | --- | --- | --- | --- | --- | --- | --- | --- | --- | --- |
| Incidence of diabetes | 18 studies (n=8,357, 727) | RCT | Some concern | | Not serious | Not serious | Not serious | Not serious | 0.81  (0.71,0.93) | Moderate |
|  |  |  |  | | I^2^=0% |  |  |  |  |  |
| Weight change | 11 studies (n=7,104) | RCT | Low | | Serious | Not serious | Not serious | Not serious | -0.62  (-1.22,-0.02) | Moderate |
|  |  |  | |  | I^2^=65% |  |  | | |  |

# Supplementary Figure 1. Risk of bias assessment for the incidence of diabetes in women with recent gestational diabetes.


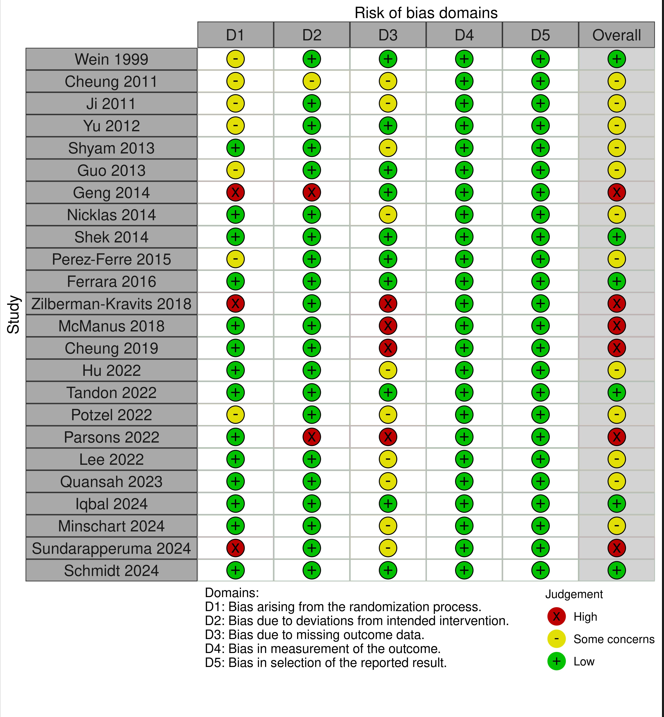


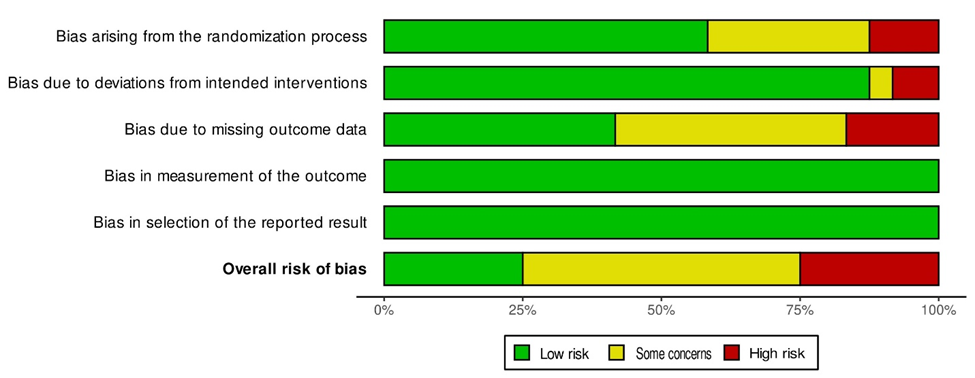


Top: Individual studies. Bottom: Overall.

# Supplementary Figure 2. Risk of bias assessment for the weight change in women with recent gestational diabetes.


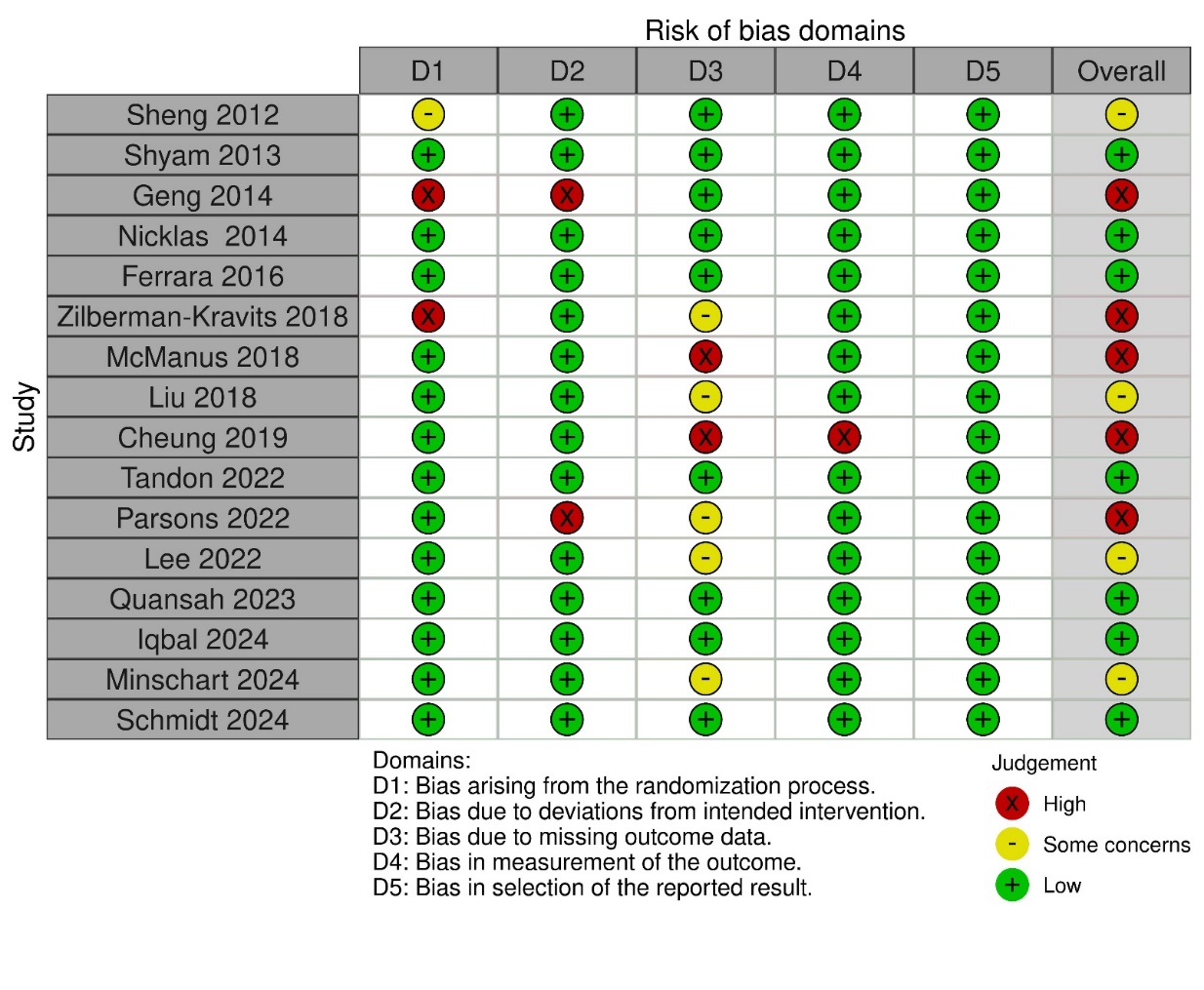


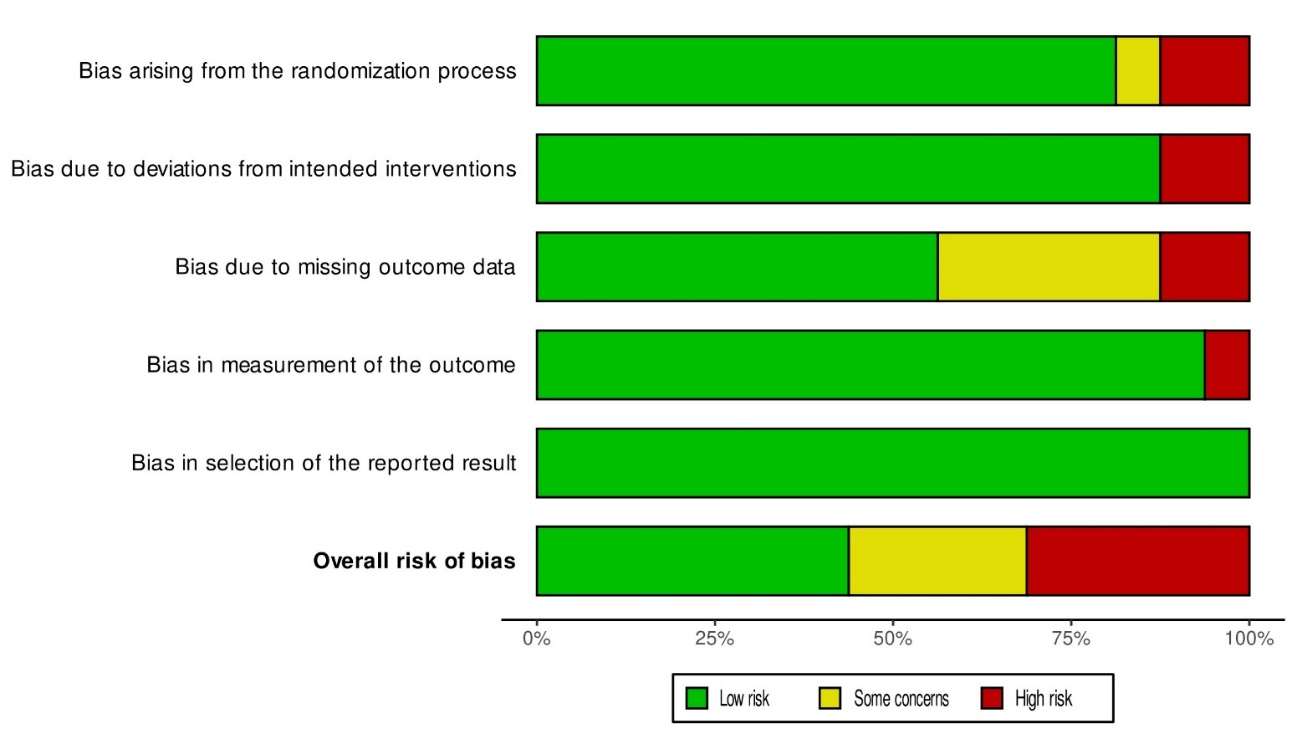


Top: Individual studies. Bottom: Overall.

# Supplementary Figure 3. Publication bias.


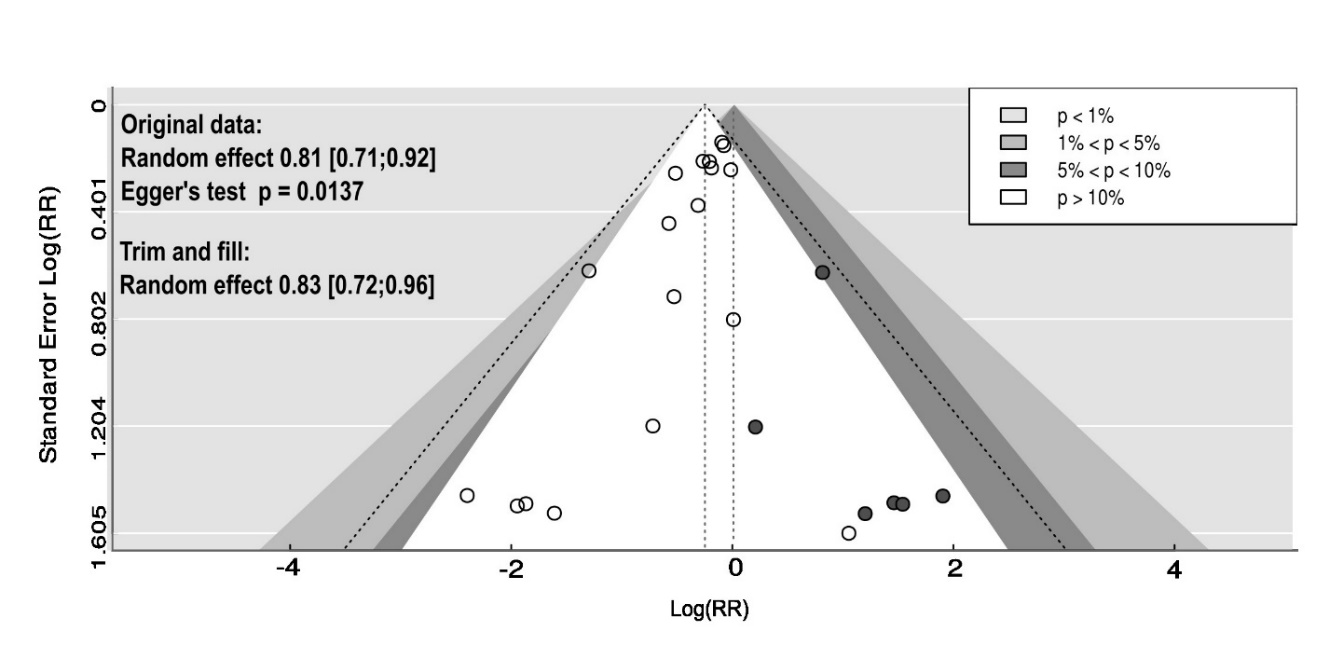


Funnel plot (white circles, 18 studies) and trim and fill (blackened circles, adding six studies) to assess publication bias regarding the incidence of diabetes in studies without high risk of bias. The black dashed line represent the 95% pseudo confidence interval, indicating the region within which we would expect 95% of studies to lie if the studies are all estimating the same underlying effect (low heterogeneity). The contour regions highlight the regions in which studies would need to be to achieve a given level of significance. If the region where studies are perceived to be missing is an area of statistical non-significance (indicated by the white area) then this adds strength to the possibility that asymmetry is caused by publication bias.
